# Protozoan Communities and Their Contribution to Predation on *E. coli* in Aerobic Granular Sludge

**DOI:** 10.1021/acs.est.5c03981

**Published:** 2025-10-27

**Authors:** Zhaolu Feng, Yi Yang, Norbert C. A. de Ruijter, Nora B. Sutton, Mark C. M. van Loosdrecht, Heike Schmitt

**Affiliations:** † Environmental Technology, 4508 Wageningen University and Research, P.O. Box 17, 6700 AA Wageningen, The Netherlands; ‡ Laboratory of Cell and Developmental Biology, Wageningen University and Research, P.O. Box 633, 6700 AP Wageningen, The Netherlands; § Department of Biotechnology, Delft University of Technology, Van der Maasweg 9, 2629 HZ Delft, The Netherlands; ∥ National Institute of Public Health and the Environment, Antonie van Leeuwenhoeklaan 9, 3721 MA Bilthoven, The Netherlands

**Keywords:** protozoan community, predation, aerobic granular sludge, sludge size, bacterivory, sessile ciliate

## Abstract

Protozoa contribute to water purification through predation in wastewater treatment systems. Full-scale aerobic granular sludge (AGS) reactors treating municipal wastewater contain AGS of varying sizes, with those larger than 2 mm dominating. These size fractions exhibit different sludge morphologies and microbial communities. To date, little is known about protozoan communities and their role in the removal of human-associated bacteria (like pathogens) in AGS plants, particularly across different size fractions. This study conducted uptake experiments with fluorescent *Escherichia coli*, as a model for human-associated bacteria, followed by microscopic observation to investigate protozoan communities and their predatory behavior in six AGS size fractions and activated sludge collected from full-scale municipal wastewater treatment plants. Sessile ciliates, particularly *Epistylis* and *Vorticella*, dominated protozoan populations across six AGS size fractions, with *Epistylis* being more abundant in larger AGS fractions (>1 mm) and *Vorticella* in smaller fractions (<1 mm). Additionally, microcosm experiments under aerobic (including predation) and anoxic conditions (excluding predation) revealed that predation was likely to be the main *E. coli* removal pathway, contributing an additional 0.5 to 2.5 log_10_ CFU mL^–1^ reduction over a combination of non-predatory biological and abiotic processes. Larger AGS fractions showed greater predation capacity, linked to higher *Epistylis* abundance, while activated sludge, dominated by *Vorticella*, resembled smaller AGS fractions with lower predation capacity. These findings advance the understanding of the distribution of protozoan communities and their contribution to *E. coli* removal by predation in AGS wastewater treatment.

## Graphical abstract

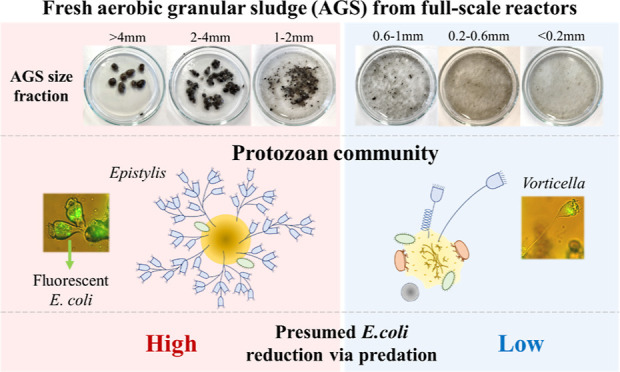

## 1 Introduction

Protozoa are ubiquitous in natural aquatic environments, where they play an essential role in the aquatic food web, and also thrive in artificial ecosystems such as wastewater treatment plants (WWTPs).^1–4^ Various protozoan species have been observed in conventional activated sludge WWTPs.^3,5–8^ Among them, ciliates, including sessile, crawling, and free-swimming types, often dominate in both biomass and species diversity in aerated activated sludge tanks, with densities reaching approximately 10^7^ cells L^–1^.^4^ Due to their predatory behavior, ciliates are involved in the removal of suspended bacteria and particulate matter, contributing significantly to water purification.^9^

In activated sludge WWTPs, the protozoan community composition is not static but responds dynamically to operational parameters. For example, previous studies have reported a shift in dominance from free-swimming to sessile ciliates with increasing sludge retention time (SRT),^10^ while higher organic matter loading has been associated with reduced protozoan diversity.^1^ In addition to operational parameters, different sludge morphologies in biological WWTPs also shape varying protozoan communities. The formation of surface-attached biofilms, multicellular aggregates of single-celled organisms, is considered a bacterial strategy to resist predation.^11,12^ This process may select for protozoa with stronger predatory capacities, resulting in protozoan communities within biofilms that differ from those found in floc-based activated sludge.

Aerobic granular sludge (AGS), a dense and compact form of spherical biofilm, is considered an alternative to activated sludge for wastewater treatment due to its efficient settling properties and suitability for compact reactor designs.^13,14^ In the full-scale AGS reactor, larger granules (>2 mm) typically predominate, while medium (0.2–2 mm) and small (<0.2 mm) size fractions coexist.^15^ These AGS size fractions exhibit various sludge morphologies and microbial communities.^16^ Previous studies on AGS granulation have shown that protozoan communities shift during the transition from seed flocculent sludge toward mature granules.^17^ However, in full-scale reactors, different AGS size fractions are subject to varying SRTs, and their distribution fluctuates dynamically due to operational factors such as excess sludge discharge and the introduction of influent suspended solids. This variability suggests that current lab-scale observation of protozoan community changes during AGS granulation under stable conditions can not reflect the community composition across different size fractions in full-scale reactors. Therefore, further investigation into protozoan communities within fresh AGS fractions of varying sizes, collected from full-scale reactors under real operational conditions, is needed.

Some protozoa exhibit selective predation on bacteria,^11,12^ thereby shaping bacterial communities to some extent in WWTPs.^18^ Since pathogens are a subset of the bacterial population in wastewater, their ingestion by protozoa contributes to the pathogen reduction and helps mitigate waterborne diseases. Indeed, previous studies on activated sludge systems have found a reduction in viable *Escherichia coli* abundance in the presence of protozoa, and certain ciliated protozoa, such as *Vorticella* and *Aspidisca*, were observed preying on fluorescent *E. coli*.^8,19^ In addition, Barrios-Hernández et al.^20^ found enhanced *E. coli* removal in a lab-scale AGS reactor where protozoa were present. However, these findings are primarily based on conventional activated sludge systems or reflect only the overall removal performance in the AGS reactor. Thus, the potential contribution of protozoan predation to *E. coli* removal across different AGS size fractions in full-scale AGS systems remains unclear. Gaining such insight could support the development of feasible strategies, such as optimizing the distribution of AGS size fraction, to further enhance *E. coli* removal.

The main goal of this study was to investigate protozoan community composition and their predation potential across six AGS size fractions in a full-scale plant. *E. coli* was used as a model organism for pathogens. First, uptake experiments were performed by spiking fluorescent *E. coli* into sludge samples, followed by microscopic observations using both fluorescence and brightfield microscopy. These observations aimed to assess the distribution of protozoan communities across AGS size fractions and evaluate their predatory behavior. Second, two microcosm experiments were conducted under aerobic and anoxic conditions to evaluate the potential contribution of predation to viable *E. coli* removal in each size fraction. In our microcosm, *E. coli* removal can occur through multiple pathways, including protozoan predation, abiotic processes like attachment, and non-predatory biological interactions like antagonism from indigenous bacteria or bacteriophage-mediated lysis.^21–23^ To isolate predation effects, aerobic (with predatory behavior) and anoxic (without predatory behavior) conditions were used. Pre-experiments confirmed that protozoan activity was effectively suppressed under anoxic conditions. Finally, similar uptake and microcosm experiments were conducted using activated sludge to compare protozoan communities and their contributions to *E. coli* removal between AGS and activated sludge. Overall, our results offer new insights into the protozoan community and its potential contributions to *E. coli* removal in AGS wastewater treatment.

## 2 Materials and Methods

### 2.1 Sampling of AGS and Activated Sludge and AGS Sieving

AGS samples were collected from a full-scale AGS plant in Utrecht, The Netherlands (Figure S1), which treats municipal wastewater for 430,000 population equivalent.^24^ Six circular AGS reactors operate in fed-batch mode for organic carbon and nutrient removal. Activated sludge samples, typically smaller than 0.2 mm in size, were obtained from a WWTP in Bennekom, The Netherlands (Figure S1), serving 35,000 population equivalent. Detailed operational and water quality parameters are presented in Table S1.

To ensure AGS samples were representative of the entire AGS plant, mixed liquor samples (containing both sludge and liquid) were pooled from various locations and depths across multiple reactors during the first 15 min of aeration (Text S1). Additionally, 5 L of mixed liquor activated sludge samples was collected from the conventional WWTP in Bennekom, The Netherlands (Figure S1). All samples were transported to the laboratory within 4 h, sieved on the same day to obtain six AGS size fractions: >4 mm, 2–4 mm, 1–2 mm, 0.6–1 mm, 0.2–0.6 mm, and <0.2 mm, and resuspended in original supernatant from AGS reactor and maintained under continuous aeration for microscopic uptake observation. The wet sieving procedures used to prepare size fractions for microscopic uptake observations and microcosm experiments are described in detail in Text S2 and Table S2. According to pre-experimental results obtained from settled-only sludge (Text S3 & Figure S2), this sieving process does not significantly affect the distribution of protozoan communities, especially the distribution of non-sludge-associated protozoa, across AGS size fractions.

### 2.2 Microscopy on Fluorescent *E. coli* and Protozoa

*E. coli* ATCC 25922 was selected as a model organism for Gram-negative fecal pathogens. The protocol of *E. coli* culture and enumeration is shown in Text S4 and Table S3. Bacterial fluorescent staining was used to differentiate protozoan genera capable of ingesting *E. coli* from those that are not. An overnight-grown *E. coli* ATCC 25922 suspension was stained with dsGreen 10,000× (Lumiprobe, Germany) for 15 min in the dark. The *E. coli* staining procedure was modified from a previous study (Text S5).^25^

Uptake experiments to investigate predatory behavior on fluorescent *E. coli* were conducted in a 24-well plate (Figure 1A). Each well contained a specific amount of sludge fraction (Table S4), into which fluorescent *E. coli* cells were added, resulting in a working concentration of 1 × 10^6^ CFU mL^–1^ after gentle mixing. This level is within the in situ *E. coli* concentration range (10^5^–10^7^ CFU mL^–1^) detected in the influent of the target AGS plants over one year to reflect natural prey availability while assessing the presence or absence of protozoan predation. The mixed sludge samples were incubated in the dark at room temperature for 15 min. Following incubation, ice-cold paraformaldehyde (PFA) at 1.6% final concentration was added to the wells to deactivate and immobilize microorganisms without lysing or damaging their internal structure (Text S6 & Figure S3), facilitating the subsequent microscopic recognition of protozoan genera.

**Figure 1:**
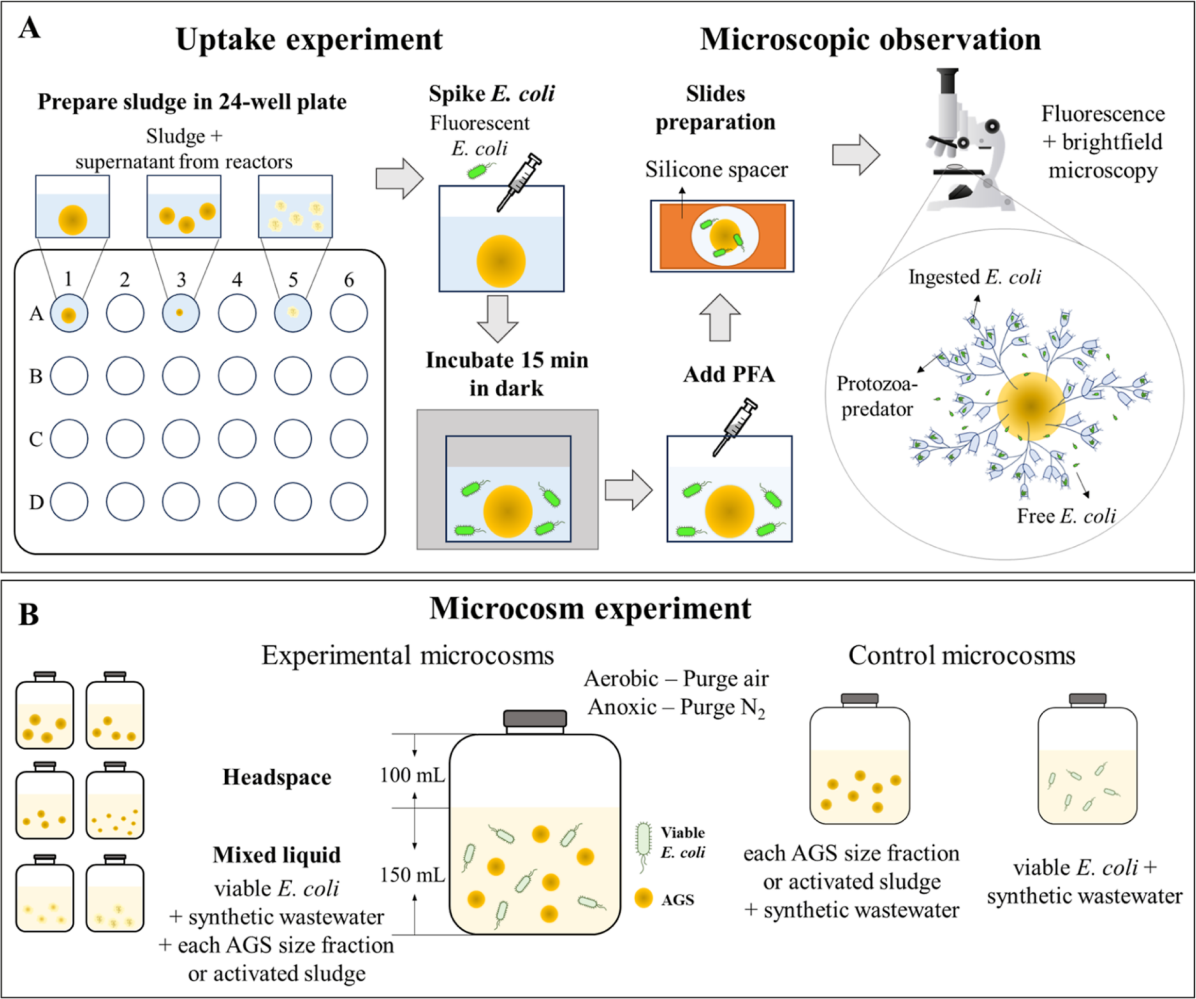
Schematic diagram of uptake experiments, microscopic observation (A), and batch microcosm experiments (B).

Sludge samples were prepared on microscope slides with silicone spacers (Grace FastWell^TM^) for microscopic observation of sludge in fixed volumes. Three slides per sludge fraction were analyzed on a Nikon Eclipse 80i fluorescence microscope (Text S7). Protozoa were imaged in brightfield, fluorescence, and overlapping modes to differentiate the protozoan genera and determine their ability to ingest fluorescent *E. coli*. Since counting individual *E. coli* cells within the food vacuoles of the protozoa was unfeasible, each ciliate ‘head’ with fluorescent bacteria inside was counted as a distinct predator. Protozoan genera were identified based on the referenced booklist.^26^

Slight difference in amounts added and the pretreatment process for each sludge fraction due to varying sizes in uptake experiments, slide preparation, and microscopic observation were standardized (Text S7, Table S4 & Figure 1A). To assess accuracy and reproducibility of data, samples were collected three times from both AGS and the activated sludge plants between November 2023 and February 2024. At each time point, triplicate slides were prepared for each sludge fraction, with sampling dates listed in Tables S4 and S5.

### 2.3 Microcosm Experiments on *E. coli* Removal under Aerobic and Anoxic Conditions

Triplicate microcosm experiments were conducted in 250 mL batch bottles to examine *E. coli* removal across six AGS size fractions and activated sludge (Table S5). Each microcosm contained sludge mixed with synthetic wastewater, achieving a sludge concentration of approximately 6 g suspended solids per liter (g SS L^–1^), representing the biomass concentration (Figure 1B).

Microcosm experiments were conducted under aerobic or anoxic conditions. In the experiment under aerobic conditions, *E. coli* reduction was expected to involve predation, abiotic, and non-predatory biological processes. Air was purged into both the headspace and liquid phase of the batch bottles to maintain protozoan activity. Viable *E. coli* stock was spiked to achieve an initial concentration of approximately 1 × 10^5^ CFU mL^–1^. The bottles were shaken horizontally at 120 rpm and 20 °C for 24 h. Liquid samples were collected at 0, 1.5, 3, 6, 9, 21, and 24 h. After settling for 1 min, the supernatant of each sample was enumerated for *E. coli* number as described in Text S4.

Anoxic experiments, where protozoan activity was inhibited, focused on abiotic and non-predatory biological processes. Pre-experiments (Text S8) were conducted under three conditions: alkaline (by adding 13.5 g L^–1^ NaHCO_3_), low-temperature (4 °C), and anoxic conditions (by purging nitrogen gas into both headspace and liquid phases).^20,27^ Results confirmed that only anoxic conditions (by purging nitrogen gas) effectively inhibited protozoan activities for 24 h, as evidenced by the absence of predation on fluorescent *E. coli* and the lack of microscopically visible movement in experiments similar to the uptake experiments (Figures S4 and S5). Other experimental steps and sampling for anoxic microcosm experiments followed the same protocol as aerobic microcosm experiments.

Nine control microcosms were treated under the same conditions as experimental microcosms (Text S9 & Figure 1B). Seven control microcosms containing synthetic wastewater and either an AGS size fraction or activated sludge (without *E. coli*) were prepared to assess the detachment of background *E. coli* from fresh sludge. *E. coli* concentration in these control bottles was below 10^1^ CFU 100 mL^–1^, which was negligible compared to the spiked *E. coli* concentrations in experimental microcosms (10^5^ CFU mL^–1^). Two additional control microcosms containing synthetic wastewater and spiked *E. coli* (without sludge) were prepared to evaluate the stability of *E. coli* activity over 24 h, which confirms no significant inactivation, with *E. coli* concentrations remaining stable at 10^5^ ± 10^3^ CFU mL^–1^ over 24 h (scored at 0 and 24 h).

### 2.4 Statistical Analysis

The *E. coli* removal curves, represented by *C*_t_/*C*_0_, under aerobic and anoxic conditions were fitted to first-order bacterial decay models (Text S10).^28^ The assumed predation of *E. coli* was evaluated by comparing batch microcosm experiments under aerobic and anoxic conditions (Text S10). Simple linear regressions were employed to analyze the relationships between protozoan abundance and predation rates for *E. coli* in six AGS size fractions. Sludge concentration was measured using standard methods.^29^

## 3 Results and Discussion

### 3.1 Protozoan Community and Its Predatory Behavior Across Six AGS Size Fractions

Brightfield microscopic observations revealed that protozoan communities across six AGS size fractions were mainly ciliates and amoebas, including sessile ciliates (*Epistylis*, *Vorticella*, and *Opercularia*), crawling ciliates (*Aspidisca*), free-swimming ciliates (*Glaucoma* and *Trachelophyllum*), and testate amoebas (*Arcella*) (Table 1).^26^ These protozoan genera are frequently observed in biological wastewater treatment processes, and their communities have been shown to shape bacterial populations.^3,18,30,31^ Certain ciliates influence effluent suspended solid quality and have been suggested as bioindicators of contaminant removal performance in WWTPs. For example, *Vorticella* helped reduce suspended solids by ingesting biomass particles, while the abundance of *Epistylis* and *Aspidisca* correlated with ammonium and total nitrogen removal.^7,32^ *Epistylis*, acting as the skeleton of granules, also plays a vital role in the granulation processes of both AGS and anaerobic ammonium oxidation sludge.^17,33^ Despite these studies reporting certain functions of these genera in wastewater treatment, their predatory behavior in AGS systems remains understudied.

**Table 1:**
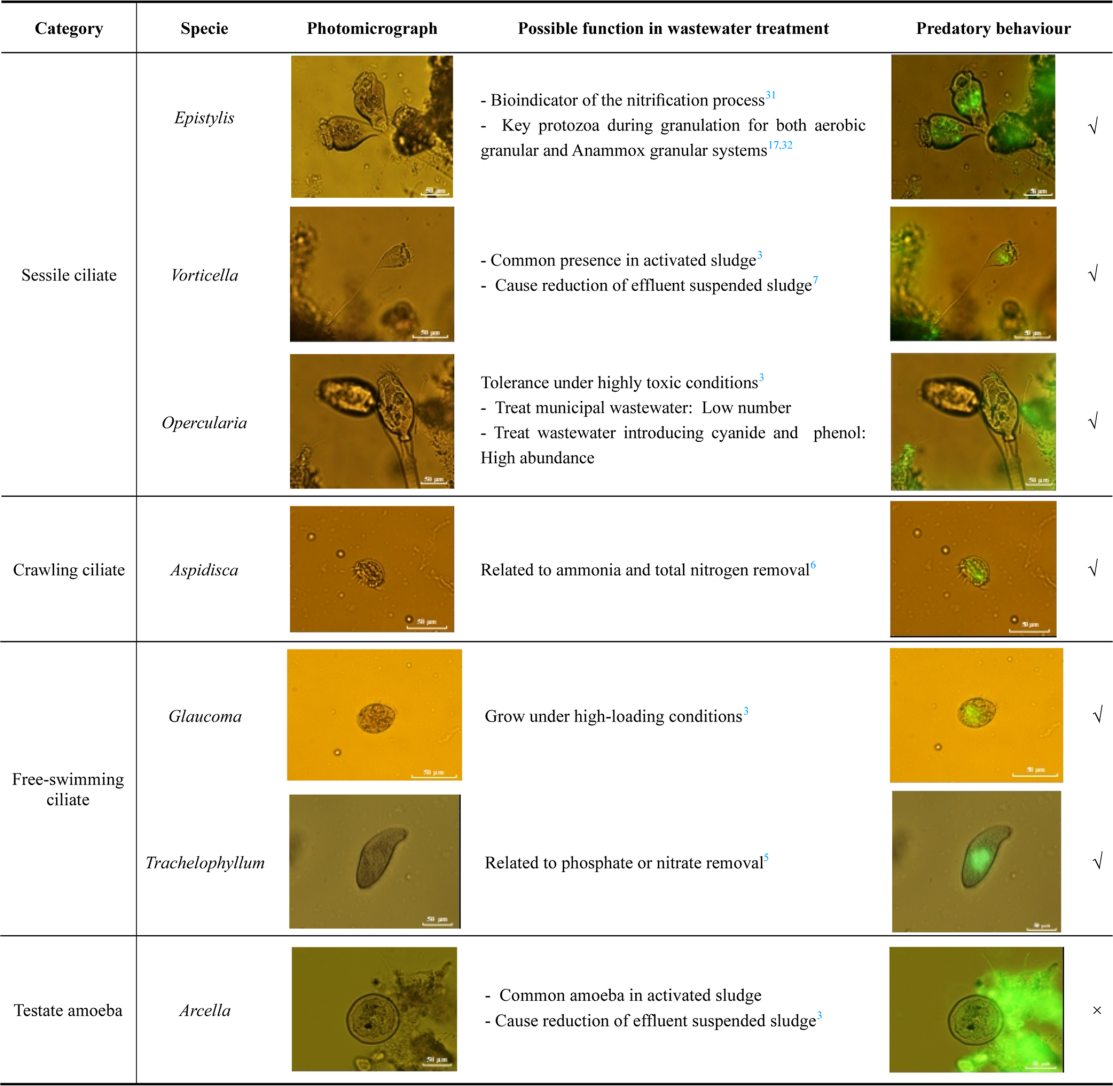
Observed Protozoan Species in Six AGS Size Fractions and Activated Sludge: Their Possible Function in Wastewater Treatment Systems and Predatory Behaviour toward *E. coli* as Identified in This Study

Protozoan predation behavior was observed through phagocytosis assays, in which protozoa engulf and internalize fluorescently stained bacteria. These uptake experiments were followed by microscopic observations to assess ingestion. Uptake was fast and abundant, and appeared to be unaffected by any potential loss of *E. coli* viability resulting from the DNA staining process. Aggregated fluorescent *E. coli* cells were frequently and abundantly identified inside the food vacuoles of six protozoan genera, showing that sessile ciliates (*Epistylis*, *Vorticella*, and *Opercularia*), crawling ciliates (*Aspidisca*), and free-swimming ciliates (*Glaucoma* and *Trachelophyllum*) could ingest *E. coli* within AGS systems (Table 1). These ciliates capture *E. coli* through a filter-feeding process that generates a water current through their cilia, capturing particles from 0.3 to 5 μm.^34,35^ Since *E. coli* cells (1 to 2 μm) fall within this range, these ciliates likely play a key role in *E. coli* removal in AGS systems.

An interesting observation was that *Glaucoma* (ciliates of the suborder *Tetrahymenina*) ingested *E. coli*, but the frequency of this predatory behavior varied across AGS size fractions. In AGS smaller than 0.6 mm, over 95% of the observed *Glaucoma* individuals ingested fluorescent *E. coli* (84 ± 2 out of 87 ± 6 individuals), whereas only 39% to 68% of the observed *Glaucoma* exhibited this behavior in AGS larger than 0.6 mm (4 ± 2 out of 13 ± 4 individuals to 30 ± 6 out of 45 ± 10 individuals), based on 1012 images across 75 slides (Figure S6). *Glaucoma*, a free-swimming ciliate, prefers environments with abundant substrate and lower competition, likely due to its relatively low efficiency in capturing suspended bacteria compared to sessile ciliates.^36^ The reduced predatory activity observed in larger fractions may be attributed to increased competition for *E. coli* from other protozoan genera, such as sessile ciliates. Similarly, no fluorescent *E. coli* was found in testate amoebas (Table 1), which may also be due to their limited competitiveness and slow movement.^3,37^

### 3.2 Protozoan Abundance in AGS

#### 3.2.1 Sessile Ciliate: Dominant Protozoa in AGS Fractions

Protozoan communities across six AGS size fractions were quantified using brightfield microscopy. Both the observed counts (individuals) and biomass-normalized absolute abundances (individuals per gram of biomass, ind g SS^–1^) were determined, with each sessile ciliate colony head counted as a single individual. Sessile ciliates dominated all AGS size fractions, with abundance ranging from 2.3 × 10^5^ to 1.4 × 10^6^ ind g SS^–1^, corresponding to 362 to 2802 observed individuals per microscopically scanned AGS/activated sludge slide (Figure 2A and Table S6).

**Figure 2:**
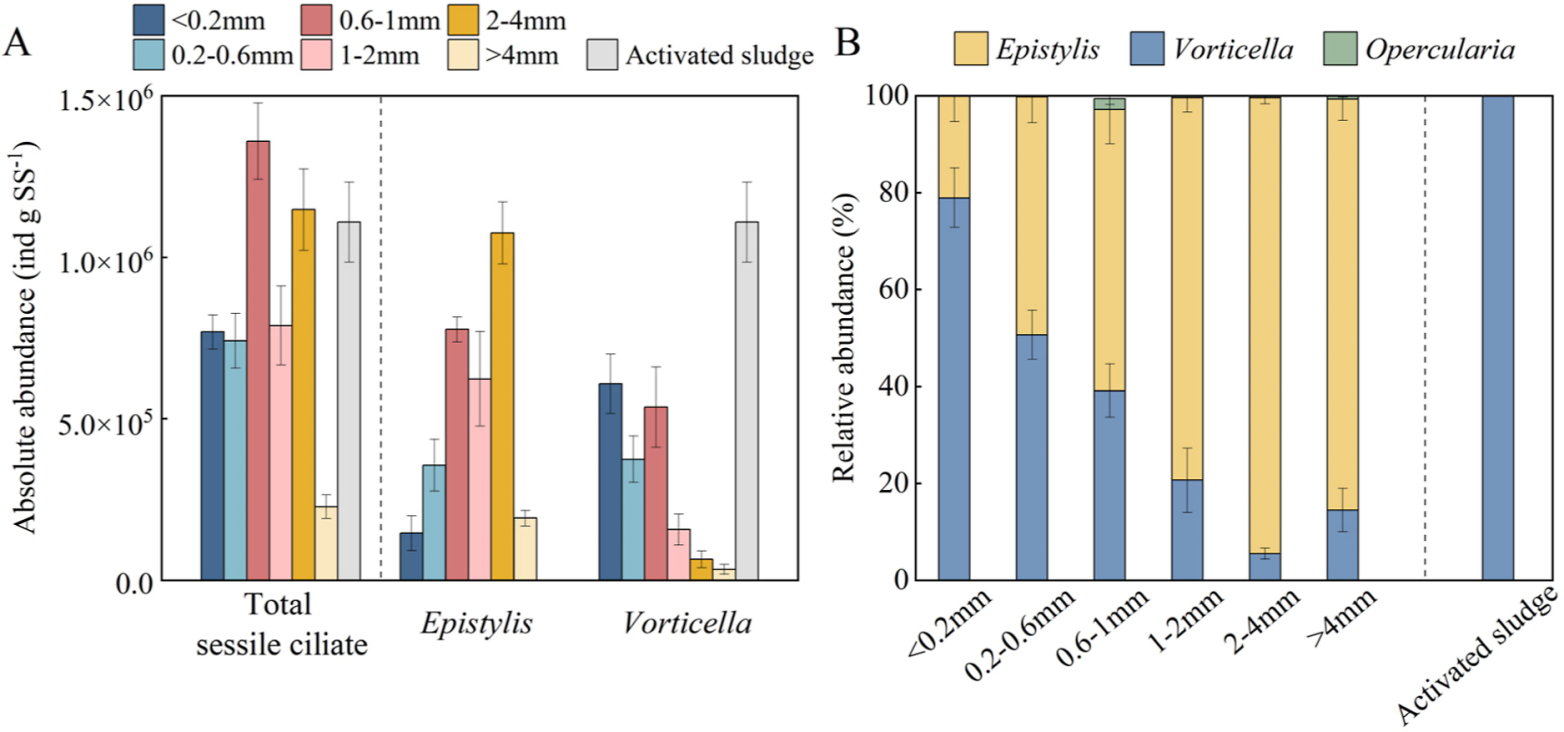
Absolute abundance of total sessile ciliate, *Epistylis*, and *Vorticella* (A); relative abundance of *Epistylis*, *Vorticella*, and *Opercularia* (B) in AGS size fractions and activated sludge; absolute abundance refers to the count of in sludge samples using microscopy, while relative abundance represents the proportion of a specific protozoan category relative to total counts of sessile ciliate. Error bars represent standard deviation based on triplicate observations. Data for activated sludge are discussed in Section 3.5.

Significantly higher counts of sessile ciliates (*p*-value <0.05) were observed in larger AGS size fractions (>1 mm), particularly within the 1–4 mm range (Table S6). When normalized by biomass concentrations (ind g SS^–1^), AGS between 0.6 to 4 mm also exhibited relatively higher absolute abundances of sessile ciliates, suggesting their preferential enrichment in this intermediate size range (Figure 2A). Interestingly, despite relatively high individual counts of sessile ciliates were observed in the largest granules (>4 mm), their biomass-normalized abundances were the lowest (ind g SS^–1^). This discrepancy is likely due to anaerobic conditions within the core of larger granules, where oxygen limitation inhibits protozoan survival. This is supported by previous findings showing that tree-like sessile ciliates preferentially attach to the outer surfaces of granules, with their heads absent from the anoxic interiors.^17^

The differential distribution of sessile ciliates across six AGS size fractions is likely driven by ecological selection within protozoan communities. During AGS granulation, protozoan dominance shifted from free-swimming ciliates in seed flocs to sessile ciliates in mature granules, as sessile ciliates play an important role in aggregate formation.^17^ Tree-like sessile ciliates, such as *Epistylis*, colonize floc surfaces, increasing the surface area for bacterial attachment. Due to their rapid growth, sessile ciliates outcompete crawling and free-swimming ciliates for particulate matter and bacteria, leading to their dominance in larger granules.^17^ As granules form, dead sessile ciliates become embedded within the matrix, serving as a skeletal framework and scaffold for living colonies.^33^ This competitive pressure may also cause free-swimming ciliates to move toward the liquid phase. In addition to ecological interactions, operational parameters such as SRTs may further shape protozoan community composition. In full-scale AGS reactors, SRTs vary significantly by AGS sizes, with reported values of approximately 3 days for <0.2 mm, 7 days for 0.2–2 mm, and 140 days for >2 mm fractions.^15^ Longer SRTs are generally associated with reduced protozoan diversity,^38^ but greater relative abundances of sessile ciliates,^10^ which likely contributes to their enrichment in larger AGS size fractions.

Additionally, protozoa not associated with the sludge surface, such as free-swimming ciliates and amoebae, showed relatively higher observed counts and biomass-normalized abundance (ind g SS^–1^) in smaller AGS size fractions (Figure S7 and Table S6). The trend is likely due to the reduced volume of supernatant available on microscope slides when analyzing larger granules, as evidenced by comparable normalized counts of free-swimming protozoa per liter of supernatant (Tables S4 and S6). As non-sludge-associated protozoa are generally assumed to be evenly distributed in the liquid/supernatant phase, the smaller supernatant volume may have limited their detection during microscopy. It is worth noting that these non-sludge-associated protozoa primarily inhabit the liquid phase of AGS reactors, where they are subject to dynamic inflow and washout. Given their transient occurrence and association with the liquid phase, these non-sludge-associated protozoa are acknowledged but not further analyzed in this study.

#### 3.2.2 *Epistylis* are Dominant Sessile Ciliates in AGS Larger than 0.6 mm

*Epistylis* dominated sessile ciliates in AGS larger than 0.6 mm, with relative abundances ranging from 57.5%–93.9%, while *Vorticella* was the second most abundant. Interestingly, *Epistylis*’s relative abundance increased with AGS size, while *Vorticella*’s decreased. The remaining sessile ciliate (*Opercularia*) constituted less than 2.3% of the total sessile ciliates across all AGS fractions (Figure 2A,B). The distinct distribution of *Epistylis* and *Vorticella* likely reflects their differing morphology. *Epistylis*, with its fixed stalks and multiple heads, provides a more stable framework for granulation, while *Vorticella*’s single head and retractable stalk may limit its role in larger AGS size fractions.^39^ This aligns with the previous findings that *Epistylis* replaced *Vorticella* as the dominant sessile ciliates during early aerobic granule formation.^40^

### 3.3 Overall *E. coli* Removal in AGS Size Fractions

Batch microcosm experiments were conducted under aerobic conditions to assess bacterial removal across six AGS size fractions. *E. coli*, a model of Gram-negative fecal pathogens, was introduced to evaluate its removal via predation, non-predatory biological processes, and abiotic processes. Over 24 h, *E. coli* reduction ranged from 1.4 to 3.3 log_10_ CFU mL^–1^, with larger AGS size fractions (>1 mm) exhibiting higher capacity and faster rates (Figure 3A). *E. coli* concentrations decreased exponentially, fitting a first-order bacterial decay model (Figure S8 and eq S1).^28^ A significant *E. coli* reduction was observed by the first measurement at 1.5 h, and concentrations gradually stabilized at around 1.1 × 10^3^ CFU mL^–1^ (or 3 log_10_) at 24 h (Figure 3A). The total *E. coli* reduction across six AGS size fractions ranged from 1.4 log_10_ (95.6%) to 3.3 log_10_ CFU mL^–1^ (99.9%) within 24 h (Figure 3B).

**Figure 3:**
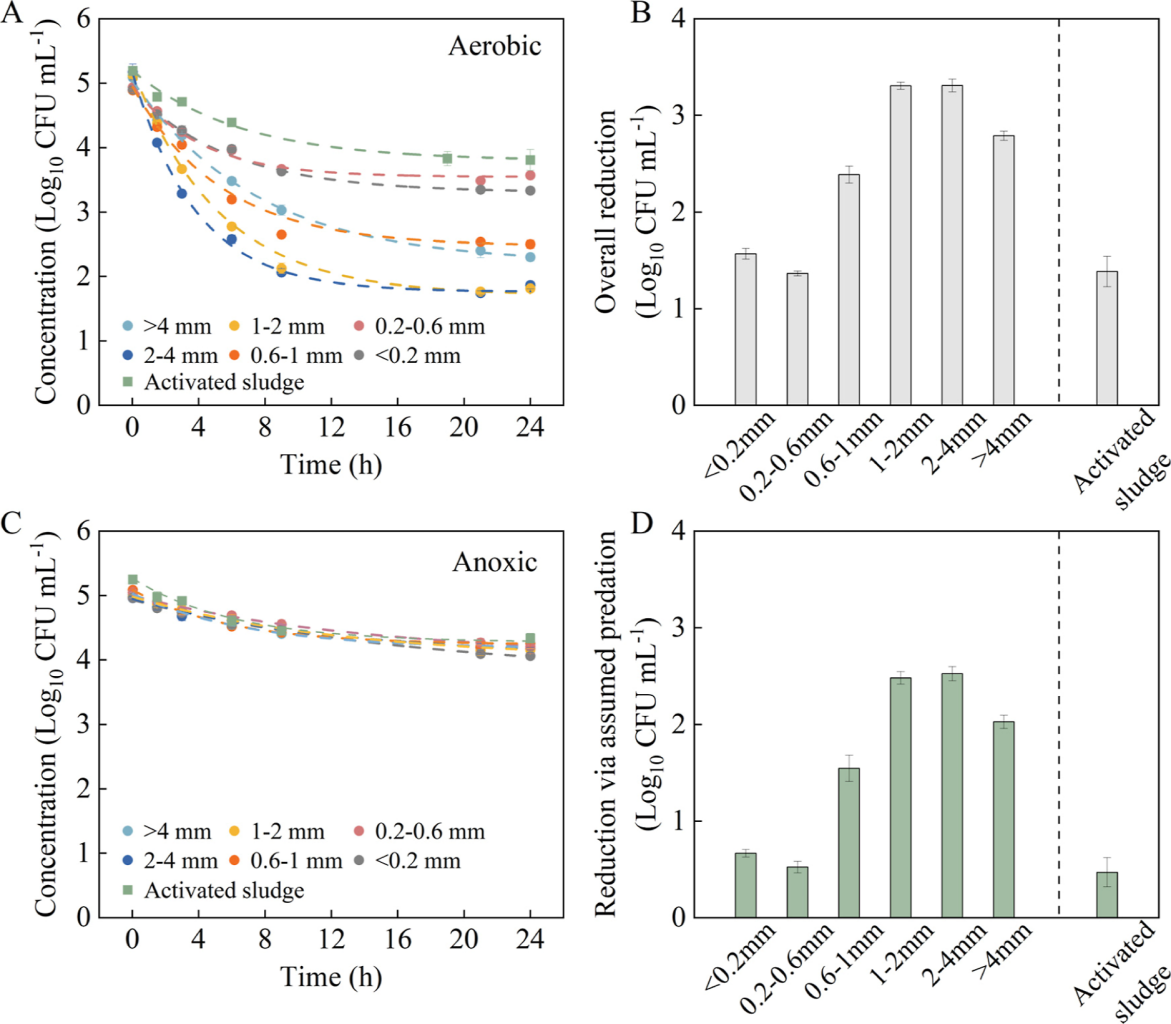
Log_10_ *E. coli* removal under aerobic conditions in batch microcosms with AGS fractions or activated sludge (A); *E. coli* reduction at 24 h under aerobic conditions attributed to predation, non-predatory biological and abiotic processes (B); Log_10_ *E. coli* removal under anoxic conditions (C); Difference in *E. coli* reduction between aerobic and anoxic conditions at 24 h, representing presumed predation (D). Error bars represent standard deviation based on triplicate experiments.

*E. coli* removal varied across six AGS size fractions. Among the three largest fractions (>4 mm, 2–4 mm, and 1–2 mm), 2.8, 3.3, and 3.3 log_10_ CFU mL^–1^ were removed, respectively, showing higher removal compared to AGS fractions between 0.6 and 1 mm (2.4 log_10_ CFU mL^–1^ removed) and AGS smaller than 0.6 mm (1.3 to 1.6 log_10_ CFU mL^–1^ removed), based on results from triplicate experiments (Figure 3B). In terms of removal capacity per gram biomass, AGS larger than 1 mm exhibited higher removal capacity, with 1.67 × 10^7^ CFU removed g SS^–1^, surpassing other fractions by 10^4^ to 10^5^ CFU removed g SS^–1^ (Table 2). A higher first-order bacterial decay rate ( $k_{1\text{\_}overall\;removal}$ ) was also observed in AGS larger than 1 mm. These results indicate that larger AGS size fractions (>1 mm) removed *E. coli* more rapidly and effectively.

**Table 2:** *E. coli* Removal Capacity and Rates through Different Processes: Overall Removal (Predation, Non-Predatory Biological Process, and Abiotic Process), Removal Excluding Predation (Non-Predatory Biological Process and Abiotic Process), and Presumed Predation across Six AGS Size Fractions and Activated Sludge

	overall removal			removal excluding predation			removal via presumed predation
sludge fraction	average capacity (CFU g SS^–1^)	Capacity compared to AGS size >1 mm^a^ (CFU g SS^–1^)	*k*_1_overall removal_ ^b^ (h^–1^)	average capacity (CFU g SS^–1^)	capacity compared to AGS size <0.2 mm^c^ (CFU g SS^–1^)	*k*_2_removal excluding predation_ ^b^ (h^–1^)	*k*_3_presumed predation_ ^b^ (h^–1^)
>4 mm	1.66 × 10^7^	-	0.89	1.38 × 10^7^	–8 × 10^5^	0.18	0.71
2–4 mm	1.67 × 10^7^	-	1.50	1.39 × 10^7^	–6.5 × 10^5^	0.21	1.29
1–2 mm	1.67 × 10^7^	-	0.97	1.42 × 10^7^	–4.1 × 10^5^	0.23	0.74
0.6–1 mm	1.65 × 10^7^	–5.5 × 10^4^	0.78	1.43 × 10^7^	–2 × 10^5^	0.25	0.53
0.2–0.6 mm	1.6 × 10^7^	–7.1 × 10^5^	0.50	1.44 × 10^7^	–2.6 × 10^5^	0.25	0.26
<0.2 mm	1.62 × 10^7^	–4.4 × 10^5^	0.49	1.46 × 10^7^	-	0.25	0.25
activated sludge	1.59 × 10^7^	–7 × 10^5^	0.45	1.46 × 10^7^	–5.3 × 10^4^	0.31	0.14

a Difference between the average *E. coli* removal capacity of the three largest AGS size fractions (1–2 mm, 2–4 mm, and >4 mm) and that of the other four smaller fractions (0.6–1 mm, 0.2–0.6 mm, <0.2 mm, and activated sludge). A negative value indicates that the *E. coli* removal capacity was higher in the larger AGS size fractions compared to the other fractions.

b Values of *k*_1_, *k*_2,_ and *k*_3_ represent the bacterial decay rates for *E. coli* removal through overall removal process, the removal process excluding predation, and the removal via presumed predation, respectively; The value of *k*_3_presumed predation_ is calculated as the difference between *k*_1_overall removal_ and *k*_2_remvoal excluding predation_.

c Difference between the average *E. coli* removal capacity of the smallest AGS size fractions (<0.2 mm) and that of the other six sludge fractions (>4 mm, 2–4 mm, 1–2 mm, 0.6–1 mm, 0.2–0.6 mm, and activated sludge). A negative value indicates that the *E. coli* removal capacity was higher in the smallest sludge fraction compared to the other fractions.

### 3.4 *E. coli* Removal through Predation

*E. coli* removal via predation was evaluated by comparing batch microcosm experiments under aerobic and anoxic conditions. Non-predatory biological and abiotic processes were assumed to be unaffected by oxygen availability within 24 h. Additionally, minimal *E. coli* inactivation was observed over 24 h (Section 2.4).

#### 3.4.1 Predation Enhances *E. coli* Removal

*E. coli* removal under anoxic conditions showed less than 1 log_10_ reduction (0.76 to 0.9 log_10_ CFU mL^–1^) across six AGS size fractions over 24 h (Figure 3C), suggesting that non-predatory biological and abiotic processes were not the main removal pathways for *E. coli*. The difference in *E. coli* reduction between the aerobic and anoxic microcosm ranged from 0.5 to 2.5 log_10_ CFU mL^–1^ (average 1.63 log_10_ CFU mL^–1^) (Figure 3D), indicating that the presence of predation significantly enhanced removal by an additional 0.5 to 2.5 log_10_ CFU mL^–1^. These findings align with a previous study showing a 0.3 log_10_ CFU mL^–1^ reduction under anaerobic conditions, with an additional 1 log_10_ CFU mL^–1^ reduction upon introducing predation under aerobic conditions.^20^ Given the relatively low *E. coli* removal and slow rate under anoxic conditions, predation is likely a major pathway for *E. coli* removal in AGS systems.

#### 3.4.2 Higher *E. coli* Uptake in Larger AGS Size Fractions

AGS ranging from 2 to 4 mm and 1–2 mm showed the highest *E. coli* removal and the greatest difference in reduction between aerobic and anoxic conditions, with an additional 2.5 log_10_ CFU mL^–1^ reduction due to presumed predation. AGS larger than 4 mm followed, removing 2 log_10_ CFU mL^–1^. Conversely, smaller AGS fractions (0.2–0.6 mm and <0.2 mm) exhibited only 0.52 and 0.67 log_10_ CFU mL^–1^ additional *E. coli* reduction, respectively (Figure 3). This suggests that AGS larger than 1 mm were more effective at removing *E. coli* through predation. A similar trend was observed in presumed predation rates ( $k_{3\text{\_}presumed\;predation}$ ), with AGS larger than 1 mm showing the highest values (0.71–1.29 h^–1^), followed by fractions between 0.6 to 1 mm (0.53 h^–1^) and AGS smaller than 0.6 mm (about 0.26 h^–1^) (Table 2). These findings indicate that predation occurs more rapidly and efficiently in larger AGS size fractions (>1 mm).

We acknowledge that our results are based on the assumption that *E. coli* removal through non-predatory biological and abiotic processes is similar under aerobic and anoxic conditions. Although oxygen-limited environments may affect *E. coli* activity and removal rate, our findings show an immediate difference in *E. coli* reduction within 1.5 h between experiments conducted under aerobic and anoxic conditions. A clear decline in *E. coli* concentrations was already observed at the first measurement (1.5 h) under aerobic conditions, consistent with the rapid predation of protozoa on fluorescent *E. coli* observed within minutes in the uptake experiment. Therefore, we conclude that predation is a rapid and major bacterial removal pathway in the AGS system, though further investigation is needed to assess the contributions of other processes under aerobic and anoxic conditions.

#### 3.4.3 *Epistylis* Abundance Positively Correlated with Predation Rate

Diverse predatory behavior toward *E. coli* was observed in six AGS size fractions, likely influenced by distinct protozoan communities. To explore the potential contributions of various sessile ciliates to *E. coli* removal, simple linear regression was applied. A positive correlation (*p*-value <0.05; *R* ^2^ > 0.5) was obtained between *Epistylis* absolute abundance (individuals per gram of biomass) and *E. coli* predation rates (Figure S9), suggesting that *Epistylis* may be the key predator of *E. coli* in the AGS system.

However, our analysis only focused on sessile ciliates, without accounting for potential contributions from free-swimming protozoa present in the liquid phase due to data limitations. Additionally, the analysis was based on protozoan abundance alone and did not consider specific predation capacities of individual protozoan genera. Variations in predatory behavior, competitive interactions, and growth conditions among different protozoan genera may influence *E. coli* predation and overall removal. Meanwhile, the presence of diverse target bacteria, not only *E. coli*, could further shape protozoan predation through selective feeding. Furthermore, the assumed predation removal in this study is likely due to protozoa, metazoa, and other higher organisms, not protozoa alone. Thus, while our findings highlight the potential importance of *Epistylis*, further investigation into the specific roles and predatory behaviors of different protozoan genera is needed, with particular attention to prey diversity beyond *E. coli*.

### 3.5 Difference between Six AGS Size Fractions and Activated Sludge

#### 3.5.1 *Vorticella* Dominates Protozoan Population in Activated Sludge

Similar to AGS, sessile ciliates dominated the protozoa population in activated sludge (Figure 2A). The distribution of the four protozoan categories in activated sludge resembled that of smaller AGS size fractions (<0.6 mm), likely due to activated sludge typically being smaller than 0.2 mm, which shares comparable properties with smaller size fractions.

*Vorticella* was the predominant sessile ciliate, accounting for nearly 100% of sessile ciliates in activated sludge (Figure 2B), aligning with previous findings.^41,42^ However, some studies also reported the presence of both *Vorticella* and *Epistylis* in activated sludge.^43,44^ The discrepancy may be due to variability in the presence of *Epistylis* compared to *Vorticella*.^45^ For example, *Epistylis* was found in less than 50% of activated sludge samples (out of 200 samples) from five WWTPs, while *Vorticella* appeared in over 80% of samples.^42^ Protozoan communities in activated sludge also fluctuate seasonally due to changes in temperature and influent characteristics.^18,46^ Although we conducted triplicate uptake experiments and microscopic observations, all sampling events were during winter. Future research should examine seasonal variations in protozoan dynamics and predation.

#### 3.5.2 Lower *E. coli* Removal in Activated Sludge

Additional batch microcosm experiments were conducted with activated sludge under aerobic and anoxic conditions. Total *E. coli* reduction was approximately 1.4 log_10_ CFU mL^–1^, with 0.9 log_10_ CFU mL^–1^ removed through presumed predation and 0.5 log_10_ CFU removed mL^–1^ through non-predatory biological and abiotic processes (Figure 3). The distribution of reductions in activated sludge was comparable to AGS smaller than 0.2 mm. Although activated sludge had a high abundance of sessile ciliates, its *E. coli* removal capacity was lower than that of AGS, likely due to differences in the dominant sessile ciliates. This supports the finding that *Epistylis*, abundant in larger AGS fractions, played a more critical role in predation.

In the full-scale AGS reactor, the relative proportion of each AGS size fraction varied (Table S7). To compare *E. coli* removal between AGS and activated sludge systems, we normalized *E. coli* reduction by accounting for both the removal within each AGS fraction (CFU g SS^–1^) and the proportional distribution of those fractions within the reactor.^24^ Overall, the AGS system showed comparable or slightly higher *E. coli* reduction (4.99 log_10_ CFU g SS^–1^) compared to activated sludge (4.98 log_10_ CFU g SS^–1^), which aligns with previous studies showing that AGS matches or exceeds the performance of activated sludge in both lab-scale and full-scale setups.^25,47^ Notably, *E. coli* reduction in the AGS reactor appeared to be strongly influenced by the distribution of AGS size fractions, suggesting that optimizing AGS size distribution (e.g., increasing larger AGS size fractions) may further enhance *E. coli* removal. However, our estimation was based on the calculated removal of individual AGS size fractions and their size distribution, without accounting for potential competition or synergistic effects among different size fractions under real mixing conditions. Thus, future studies should focus on evaluating *E. coli* removal in lab-scale reactors containing mixed AGS size fractions, or directly within full-scale AGS systems operating under realistic hydraulic and operational conditions.

This study investigated protozoan communities and their predation potential in six AGS size fractions and activated sludge. In AGS fractions, sessile ciliates, particularly *Epistylis*, dominated the protozoan population in AGS larger than 1 mm. Predation enhanced bacterial removal by 0.5 to 2.5 log_10_ CFU mL^−1^(using *E. coli* as a model), compared to non-predatory biological and abiotic processes. Larger AGS size fractions (>1 mm) showed greater predation capacity, presumably due to the higher abundance of *Epistylis*. Although activated sludge had a high abundance of *Vorticella*, its predation capacity was lower than that of AGS fractions dominated by *Epistylis*. This study provides new insights into protozoan communities and their contributions to *E. coli* removal. Future studies should further confirm the relative contributions of predation and other *E. coli* removal processes, as well as the specific role of protozoan predation. Such studies could help optimize bacterial removal by adjusting sludge size distribution in AGS systems.

## Supplementary Material



## Notes

Sampling and sieving procedures (Text S1–S3); *E. coli* culture, enumeration, and staining protocols (Text S4–S5); microbial inactivation by PFA (Text S6); sludge preparation for uptake experiments and microscopic observation (Text S7); protozoan inhibition pre-experiments (Text S8); control microcosms setup (Text S9); *E. coli* removal calculation (Text S10); operational and water quality parameters of the selected full-scale plants (Table S1); composition of synthetic wastewater (Table S2); composition of tryptic soy broth for pure *E. coli* inoculation (Table S3); setup details for uptake experiments and microscopic observation (Table S4–S5); protozoan counts on slides (Table S6); AGS size distribution in full-scale AGS reactor (Table S7); schematic diagram of sampling plants (Figure S1); sieving effect on protozoan communities (Figure S2); microscopic images on inactivation by PFA (Figure S3); microscopic images on protozoan predation behavior under inhibitory conditions (Figure S4–S5); abundance of *Glaucoma* (Figure S6); abundance of free-swimming ciliates, crawling ciliates, and amoebae (Figure S7); *E. coli* removal trend (Figure S8); relationship between *Epistylis* abundances and predation rates (Figure S9) (PDF)
