# Supplementary material for: Protozoan Communities and Their Contribution to Predation on E. coli in Aerobic Granular Sludge
Source: Environ Sci Technol. 2025 Oct 27;59(44):23916–25. doi: 10.1021/acs.est.5c03981 (PMC12613806; doi:10.1021/acs.est.5c03981)
Supplement: Supplementary file 1 [file es5c03981_si_001.pdf]

Supplementary information for:

Protozoan communities and their contribution to predation on *E. coli* in aerobic granular sludge

Zhaolu Feng[a], Yi Yang[a], Norbert C A de Ruijter[b], Nora B Sutton[a], Mark C M van Loosdrecht[c], Heike Schmitt[c,d,*]

[a] *Environmental Technology, Wageningen University and Research, P.O. Box 17, 6700 AA Wageningen, the Netherlands*

[b] *Laboratory of Cell and Developmental Biology, Wageningen University and Research, P.O. Box 633, 6700 AP, Wageningen, the Netherlands*

[c] *Department of Biotechnology, Delft University of Technology, Van der Maasweg 9, 2629 HZ Delft, the Netherlands*

[d] *National Institute of Public Health and the Environment, Antonie van Leeuwenhoeklaan 9, 3721 MA Bilthoven, the Netherlands*

* Corresponding author

E-mail address: heike.schmitt@rivm.nl (Heike Schmitt)

Number of pages: 25

Number of texts: 10

Number of tables: 7

Number of figures: 9

***Text S1** Sampling of representative aerobic granular sludge (AGS) in a full-scale AGS plant*

AGS, due to its compact structure and high density, exhibits excellent settling properties, which can hinder uniform mixing during the aeration stage. To ensure that the collected AGS samples were representative of the entire AGS plant, we pooled samples from multiple locations and depths across several reactors. All mixed liquor samples were transported to the laboratory within 4 hours after sampling. Detailed information on sampling dates for microcosm and uptake experiments is shown in Table S5.

- *AGS sampling for microcosm experiments:*

Three out of the six reactors at the AGS plant were randomly selected. Within each reactor, three sampling locations were evenly spaced at quarter intervals (1/4, 1/2, and 3/4) of the reactor's diameter. At each location, 1 L of mixed liquor sample, a mixture of biomass (sludge) and liquid, was collected from three different depths (1.5 m, 4 m, and 6 m) using a specialized sampler. In total, 27 L of mixed liquor samples were collected (3 locations × 3 depths × 3 reactors) and stored in three 10-liter plastic jerrycans. Sampling was performed 15 minutes after the start of aeration to allow sufficient mixing of sludge and liquid within the reactors.

- *AGS sampling for uptake experiments involving microscopic observation:*

For the uptake experiments, which required smaller amounts of sludge for microscopic observation, mixed liquor samples were collected from three reactors, with one sampling location per reactor and three depths per location. In total, 9 L of mixed liquor was collected (1 location × 3 depths × 3 reactors) and stored in a 10 L plastic jerrycan.

***Text S2** Wet sieving of mixed liquor AGS samples from full-scale reactors*

Fresh mixed liquor AGS samples collected from multiple full-scale reactors were processed on the same day using a wet sieving procedure, following the method described by Ali et al.[(1)] The six AGS size fractions were selected in this study, including >4 mm, 2-4 mm, 1-2 mm, 0.6-1 mm, 0.2-0.6 mm, and <0.2 mm.

The selection of these specific fractions was guided by both practical distribution in full-scale reactors and different physicochemical properties. AGS in the 2-4 mm range are the most abundant in full-scale reactors, accounting for approximately 32% of the total biomass (Table S7). This size range is also considered optimal AGS performance due to the high density and excellent settling properties of the granules[(2)]. Granules larger than 4 mm tend to exhibit decreased density and a looser internal structure[(2)], making >4 mm fractions particularly relevant for assessing the potential performance limitations associated with oversized granules.

To further explore the variability of protozoan communities across granule sizes, the 0.6-1 mm and 1-2 mm fractions were also included. Previous studies have shown that granules larger than 0.5-0.6 mm often develop anoxic zones, supporting the growth of both aerobic and anaerobic microorganisms[(3-5)]. Moreover, microbial communities have been shown to differ between granules larger or smaller than 1 mm[(1)]. These insights highlight the importance of including these intermediate-size fractions in our analysis. Finally, to compare protozoan communities between AGS and activated sludge, we also included the <0.2 mm fraction, representing the typical size range of activated sludge.

- *AGS sieving for microcosm experiments:*

Each jerrycan containing 9 L of mixed liquor sample (three jerrycans, 27 L in total) was first allowed to settle for 30 minutes to separate the sludge and supernatant phases. The supernatant (typically 6-7 L, depending on sludge concentration) was carefully decanted into a clean glass beaker and reserved for later use during the sieving process.

The concentrated mixed liquor sample after settling was then subjected to a wet sieving procedure using five mesh sieves (4 mm, 2 mm, 1 mm, 0.6 mm, and 0.2 mm) to sequentially fractionate the AGS samples from the largest to the smallest size, resuting in six size fractions: >4 mm, 2-4 mm, 1-2 mm, 0.6-1 mm, 0.2-0.6 mm, and <0.2 mm.

Specifically, the concentrated mixed liquor sample was poured onto the 4 mm sieve in small portions to minimize clogging. When clogging became apparent, a small volume of previously collected supernatant was gently poured over the sludge to facilitate the passage of smaller sludge particles through the mesh. The sieve was then gently shaken to allow fine granules or flocs resting on larger granules to pass through. A small scoop was used to carefully transfer the retained sludge on the sieve into a clean glass bottle. This procedure was repeated until all the concentrated sample in the jerrycan had been processed through the 4 mm sieve. The sludge retained on this sieve was collected as the >4 mm size fraction.

The same procedure was then sequentially applied using the 2 mm, 1 mm, 0.6 mm, and 0.2 mm sieves. Due to the large amount of sludge, the supernatant originally collected from the jerrycan was insufficient to complete the sieving of all size fractions, particularly for the three smallest size fractions (<0.6 mm). Therefore, a portion of the final effluent from the Bennekom WWTP (treated municipal wastewater) was used to assist in sieving these smaller fractions for microcosm experiments. This fresh effluent was supplied via a 2 km pipeline connecting the WWTP to the laboratory. Prior to use, the effluent was settled for 30 minutes, and the supernatant was used during the sieving process.

After each sieving step, the retained sludge on the sieve was collected into separate labeled glass bottles corresponding to the following size ranges: 2-4 mm, 1-2 mm, 0.6-1 mm, and 0.2-0.6 mm. The smallest size fraction (<0.2 mm), which contained a relatively high volume of liquid due to the sieving process, was allowed to settle for approximately 10 minutes to remove a portion of the supernatant and obtain a more concentrated sludge sample suitable for storage in a 1 L glass bottle.

To prepare the sieved sludge for overnight storage before microcosm experiments, a larger liquid volume was required to ensure uniform mixing and aeration via continuous pumped air supply, and to allow for accurate measurement of sludge concentration. Since much of the original supernatant was lost during sieving, synthetic wastewater medium was used to resuspend the sludge. This synthetic medium contained typical concentrations of nutrients and organic carbon found in municipal wastewater: 50 mg L^{-1} of ammonia and 150 mg L^{-1} of sodium acetate (Table S2). All sieved sludge samples were stored in glass bottles and aerated overnight (less than 16 hours, from 18:00 to 10:00 the next morning) to maintain microbial activity prior to use in the experiment.

- *AGS sieving for uptake experiments involving microscopic observation:*

To minimize potential impacts of the sieving process on the protozoan community distribution across the six AGS size fractions, only the supernatant originally collected from the jerrycan was used during sieving, rather than the final effluent from the WWTP. This approach was feasible because uptake experiments involving microscopic observation required smaller amounts of sludge, and the available supernatant from the jerrycan was sufficient to complete the sieving process for all size fractions.

Additionally, a pre-experiment evaluating the impact of wet sieving on protozoan community distribution across AGS size fractions showed that the manual wet sieving process did not significantly alter community composition. Details of this pre-experiment are provided in Text S3. The subsequent sieving procedure followed the same steps as described for the microcosm experiments.

After sieving, the sludge of each fraction was transferred into 0.25 L glass bottles and resuspended using the original supernatant from the jerrycan, because only a small liquid volume (<0.15 L) was needed per bottle. Same with the microcosm experiments, all sieved sludge samples were aerated overnight in glass bottles using aeration pumps to simulate conditions in the full-scale AGS reactor and to maintain all biological activity.

Text S3 *Impact of wet sieving on protozoan community distribution across six AGS size fractions*

A pre-experiment was conducted to assess whether manual wet sieving alters the distribution of protozoan communities across six AGS size fractions. In this test, fresh mixed liquor AGS samples collected from full-scale AGS reactors were directly used for microscopic uptake observation instead of after wet sieving.

To obtain the size-separated AGS fractions, the mixed liquor sample was allowed to settle for 15 minutes. Three sludge fractions with different sizes were then sampled from the bottom, middle, and top of the settled volume using a plastic pipette, corresponding approximately to >2 mm, 0.6-2 mm, and <0.6 mm size fractions. The average sludge diameters of these fractions, measured via optical microscopy, were 3.8 mm, 1.2 mm, and 0.4 mm, respectively. Subsequently, the same procedures as used in the formal uptake experiments, slide preparation, and microscopic observation were applied to count the number of protozoa, including sessile ciliates, free-swimming ciliates, crawling ciliates, and amoebae, across the three fractions.

The relative abundance of sessile ciliates was 97%, 91%, and 66% in the >2 mm, 0.6-2 mm, and <0.6 mm size fractions, respectively (Fig. S2). The absolute and relative abundances of free-swimming ciliates, crawling ciliates, and amoebae, which are typically not associated with the sludge surface, were comparable between the corresponding AGS size fractions in the pre-experiment (without wet sieving) and the formal experiment (with wet sieving). These results suggest that the manual wet sieving procedure did not significantly alter protozoan community distribution, especially the distribution of non-sessile protozoa, across AGS size fractions.

Text S4 *E. coli culture and enumeration using membrane filtration*

A freeze-dried pure culture was rehydrated with 1 mL tryptic soy broth (NutriSelect® Basic) (Table S3) and cultured overnight on a shaker at 37℃ and 120 rpm. To maintain proliferating *E. coli*, the overnight culture was diluted 10-fold into fresh broth medium for an additional hour of incubation. This process produced an *E. coli* stock solution of approximately 1×10^8 Colony Forming Units (CFU) mL^{-1}. Viable counts were determined using membrane filtration[(6)].

Liquid samples were serially diluted 10-fold using the phosphate-buffered saline solution. To obtain countable colonies, we prepared a dilution series based on a factor of 10, followed by filtering 1 mL or 3 mL of diluted samples onto a 0.45 μm sterile membrane (ME

25/21 ST, WhatmanTM, Cytiva, Germany). Subsequently, the membrane was transferred onto a Tryptone Bile X-glucuronide agar plate (NutriSelect® Plus, Merck KGaA, Germany) and incubated at 37°C for 24 hours, after which blue-green colonies indicating *β*-glucuronidase positive *E. coli* colonies were counted. To ensure accuracy and reliability in counting, we cultured four diluted samples (e.g., 10^1, $10^{1.5}$, 10^2, and $10^{2.5}$) from each sample.

Text S5 *E. coli staining using dsGreen 10,000x*

A proliferating *E. coli* culture in the log phase, cultured for an additional hour, was stained using dsGreen stock. We diluted dsGreen 100x in 2 mL of *E. coli* to achieve a final concentration of 1×10^6 CFU mL^{-1}. After vortexing the fluorescent *E. coli* solution three times for 1 second each, it was incubated in the dark at room temperature for 15 minutes. Following incubation, the fluorescent *E. coli* solution was centrifuged at 8,000 rpm for 5 minutes, and the supernatant was discarded. We then added 400 µL of 1x phosphate-buffered saline (PBS) solution to the tube and gently resuspended the pellet with a pipette. This centrifugation and washing step was repeated three times to remove as much background stain as possible. On the fourth repetition, after adding 400 µL of 1x PBS solution and resuspending the pellet, an additional 1,600 µL of 1x PBS solution was added to achieve a final volume of 2 mL.

Text S6 *Immobilization and inactivation of organisms by paraformaldehyde (PFA)*

A pre-experiment was conducted to assess the effectiveness of PFA in immobilizing and inactivating organisms. In this test, 100 µL of an 8% PFA solution was added to 400 µL of the sludge samples, resulting in a final PFA concentration of 1.6%. After 15 minutes, the sludge samples were mounted with 1 mm silicone spacers to standardize the enclosed volume and were then analyzed with fluorescence microscopy. The results indicated that fluorescent *E. coli* was not taken up by organisms, such as protozoa, and that all organisms ceased moving, demonstrating that 1.6% PFA effectively immobilized and inactivated organisms (Fig. S3).

Text S7 *Sludge preparation for uptake experiments, slide preparation, and microscopic observation*

For the three large AGS size fractions (>4 mm, 2-4 mm, and 1-2 mm), either 1 or 3 granules were used for the uptake experiment to fit the volume of the wells and slides in the enclosed wells on the slides. For the other three AGS fractions (0.6-1 mm, 0.2-0.6 mm, and <0.2 mm) and activated sludge with smaller sizes, 0.4 mg mL^{-1} of mixed liquid and sludge

samples were placed in the wells (see details of sample volume and concentrations in Table S4). The quantification was corrected for differences in the biomass content of different slides.

Slides were prepared for microscopic observation of smaller AGS fractions and activated sludge using one layer of silicone isolators (Grace FastWell™, elliptical well, 32 mm in major axis, 19 mm in minor axis, 1 mm depth, 478 μL volume), while slides for larger granules were made with two layers of silicone isolators. Gentle squeezing was applied to granules larger than 4 mm to minimize squeezing. Gentle pressure was applied to granules larger than 4 mm to ensure the slides were properly mounted in the volume under the coverslip (32 mm in major axis, 19 mm in minor axis, 1 mm depth, 956 μL volume).

Sludge samples were analyzed using 20x and 40x Plan Fluor objective on a Nikon Eclipse 80i fluorescence microscope equipped with a Uvico-VIS (RappOpto Electronics) light source, a 5 Mpx DsFi1 color camera (Nikon), and a FITC filter (Chroma 31001). For the microscopic observation of large granules, mounted in 2 mm, both the front and back sides of the granules were analyzed, since the focus depth was limited to 1 mm. After observing the front side, the coverslip was gently lifted, and the flattened granule was flipped over. Once repositioned, a new coverslip was used to observe the back side of the granules.

In summary, the same surface area of six AGS size fractions and activated sludge was scanned, and all protozoa and ingested fluorescent bacteria were counted and normalized per gram of biomass. Since normalization was made for sample thickness (spacer) and biomass, while identical image acquisition settings were applied to all samples, the protozoan abundance and protozoa with ingested fluorescent bacteria could be semi-quantitatively scored.

Text S8 *Inhibition of protozoan activities under alkaline, low temperature, or anoxic conditions*

We examined the inhibition of protozoan activities under three conditions: (i) alkaline conditions by adding 13.5 g L^{-1} $NaHCO_3$, (ii) low temperature at 4°C, and (iii) anoxic conditions by purging nitrogen gas. Additional batch bottles containing each AGS size fraction or activated sludge with synthetic wastewater were prepared for each condition and shaken at 120 rpm for 24 hours. Sludge samples (2 mL) were taken at 1, 4, and 24 hours from each bottle. Fluorescent *E. coli* were spiked into the sludge samples to determine whether protozoa were still active based on their uptake behaviour of fluorescent *E. coli*.

The pre-experiment results were as follows: (i) Alkaline conditions: protozoan activities were not fully inhibited, as evidenced by the continued predation of some protozoa on fluorescent *E. coli* after 24 hours (Fig. S4A). (ii) Low-temperature conditions: After shaking

the bottles at 4°C for 1 hour, no protozoa preyed on the fluorescent *E. coli*. However, some protozoa exhibited *E. coli* uptake at 4 and 24 hours (Figs. S4B, S4C & S4D), suggesting that certain organisms, highly adaptable to environmental changes, recovered their ability to prey on *E. coli* after adapting to low-temperature conditions. (iii) Anoxic conditions: Complete inhibition of protozoan activities was observed within 24 hours, indicating that anoxic conditions effectively inhibit their activity (Fig. S5).

Text S9 *Setup of control microcosms containing synthetic wastewater with E. coli or sludge fractions.*

The sludge collected from full-scale WWTPs contained a background level of *E. coli*. Since bacterial cells can rapidly attach to physical surfaces through van der Waals, electrostatic, and hydrophobic forces[(7,8)]. Once irreversibly attached, these cells aggregate to form small flocs or become part of larger granules[(9,10)]. To assess whether this background *E. coli* detached from the sludge phase and transferred into the liquid phase, seven control microcosms were prepared containing synthetic wastewater and either an AGS size fraction or activated sludge without additional *E. coli* spiking. Additionally, to evaluate the stability of *E. coli* activity during the experiment, two additional control microcosms were prepared with synthetic wastewater and spiked *E. coli*. These nine control microcosms were shaken alongside the experimental microcosms for 24 hours.

Liquid samples from control microcosms were collected at 0 and 24 hours. After allowing the samples to settle for 1 minute, the supernatant was collected and enumerated as described in Text S2.

The concentration of *E. coli* detached from the sludge in the seven control microcosms was below 10^1 CFU $100mL^{-1}$. In the control microcosms with small AGS size fractions (< 0.6 mm) and activated sludge, *E. coli* concentrations in the liquid phase were below the detection limit (colonies < 10; Filter 5 mL of supernatant from batch bottles). These results indicate that *E. coli* detachment from the sludge can be disregarded in the microcosm experiments. Furthermore, in two control microcosms with spiked *E. coli* and synthetic wastewater, the concentration of viable *E. coli* remained stable at 0 and 24 hours (approximately 1×10^5 CFU mL^{-1}), indicating no significant inactivation of *E. coli* during the 24-hour experiment period.

Text S10 *E. coli removal curves for microcosm experiments under aerobic and anoxic conditions in the 250 mL batch bottle, as well as the calculation of assumed predation.*

The *E. coli* removal curves, represented by C_t/C_0, from two microcosm experiments were fitted using the first-order bacterial decay model[(11)].

$$C_t/C_0 = C_e/C_0 + A \times e^{-k \cdot t} \quad \text{(S1)}$$

Where C_0, C_e, and C_t (CFU mL^{-1}) are the initial concentration, equilibrium concentration, and the concentration at time t of *E. coli*, respectively; k ($hour^{-1}$) represent the rate constant of *E. coli* removal; t (hours) is the exposure time between *E. coli* and sludge; A is a constant that represents the amplitude of the exponential decay.

Assumed predation removal = Removed_ aerobic - Removed_anoxic (S2)

Where assumed predation removed (log_{10} CFU mL^{-1}) estimates the potential contribution of protozoan predation to *E. coli* reduction over the 24-hour period; Removed_ aerobic and Removed_anoxic (log_{10} CFU mL^{-1}) represent the observed *E. coli* reduction after 24 hours in microcosm experiments conducted under aerobic and anoxic environments, respectively.

Table S1. *Operational and water quality parameters of the full-scale AGS plant in Utrecht and the activated sludge plant in Bennekom. Data were obtained from the operators of both plants.*

	**Full-scale AGS plant in Utrecht**	**Full-scale activated sludge plant in Bennekom**
**Operational parameter**		
Hydraulic retention time (hour)	35 ± 5	19 ± 3
Sludge retention time (day)	3 - 60 days<br>Small AGS size fractions≈3 days;<br>Larger granules≈60 days	17 days
**Water quality parameter - Influent**		
Chemical oxygen demand (mg L^{-1})	629 ± 160	716 ± 134
Biological oxygen demand (mg L^{-1})	277 ± 74	293 ± 72
Kjeldahl nitrogen (mg L^{-1})	71 ± 5	73 ± 13
Total nitrogen (mg L^{-1})	71 ± 5	73 ± 12
**Water quality parameter - Effluent**		
Chemical oxygen demand (mg L^{-1})	27 ± 4	27 ± 6
Biological oxygen demand (mg L^{-1})	3 ± 2	2 ± 1
Kjeldahl nitrogen (mg L^{-1})	1 ± 1	4 ± 5
Total nitrogen (mg L^{-1})	4 ± 2	6 ± 6

Table S2. *Composition of synthetic wastewater.*

**Macro nutrients (mg L^{-1})**			
$CaCl_2{\cdot}2H_2O$	14	$MgSO_4{\cdot}7H_2O$	54
KCl	36		
**Phosphate buffer (mg L^{-1})**			
$Na_2HPO_4{\cdot}2H_2O$	1090	KH_2PO_4	53
**Trace elements (mg L^{-1})**			
$FeCl_2{\cdot}4H_2O$	1.2	$CoCl_2{\cdot}6H_2O$	1.2
$MnCl_2{\cdot}4H_2O$	0.3	$CuCl_2{\cdot}2H_2O$	0.018
$ZnCl_2$	0.03	H_3BO_3	0.03
$(NH_4)_6Mo_7O_{24}{\cdot}4H_2O$	0.054	$Na_2SeO_3{\cdot}5H_2O$	0.06
$NiCl_2{\cdot}6H_2O$	0.03	EDTA (Triplex II)	0.6
Na-Resazurin	0.3	HCl 36%	0.0006
**Special nutrients (mg L^{-1})**			
NH_4Cl	50	$CH_3COONa{\cdot}3H_2O$	150

Table S3. *Composition of tryptic soy broth for pure E. coli inoculation.*

**Component**	**CAS number**	**Concentration (g L^{-1})**
Tryptone	91079-40-2	17
Soytone	91079-46-8	3
Dextrose	50-99-7	2.5
NaCl	7647-14-5	5
K_2HPO_4	7758-11-4	2.5

Table S4. *Biomass amount and concentration of six AGS size fractions and activated sludge used for uptake experiments and microscopic observation.*

		Uptake experiment (in the well)				Microscopic observation (on the slide)			
Sludge fraction		Granule number	Mixed liquid in the well (mL)	Biomass conc. (mg mL^{-1})	Biomass amount (mg)	Mixed liquid on the slides (mL)[a]	Biomass conc. (mg mL^{-1})	Biomass amount (mg)	Supernatant volume on the slides (mL)[b]
AGS	>4 mm	1	1	2.1 ± 0.25	2.1 ± 0.25	0.9	2.34 ± 0.28	2.1 ± 0.25	0.24
	2-4 mm	1	1	0.83 ± 0.08	0.83 ± 0.08	0.9	0.92 ± 0.09	0.83 ± 0.08	0.3
	1-2 mm	3	0.4	2.41 ± 0.33	0.96 ± 0.13	0.9	2.41 ± 0.33	0.96 ± 0.13	0.2
	0.6-1 mm	-	0.4	0.5	0.2	0.4	0.4	0.16	0.36
	0.2-0.6 mm	-	0.4	0.5	0.2	0.4	0.4	0.16	0.36
	<0.2 mm	-	0.4	0.5	0.2	0.4	0.4	0.16	0.36
Activated sludge		-	0.4	0.5	0.2	0.4	0.4	0.16	0.36

[a] Total volume on the slides, including sludge, PFA, and supernatant (the supernatant derived from full-scale reactors).
[b] Volume of supernatant from full-scale reactors on the slides, containing some non-sessile ciliates.

Table S5. *Number of microcosm experiments conducted in 250 mL batch bottles and uptake experiments performed in the 24-well plate.*

**Microcosm experiments in batch bottles (250 mL)**			
Experimental microcosms			
Sampling time	Three times for triplicate	Different AGS size fractions	Activated sludge
		October 16rd<br>October 30th<br>November 6th	November 20th<br>November 27th<br>December 4th
Number of experimental microcosms per sampling time	Oxic<br>- purge air	6 microcosms (sizes)	1 microcosm
	Anoxic<br>- purge nitrogen gas	6 microcosms (sizes)	1 microcosm
Control microcosms			
Sampling time	Once	Different AGS size fractions	Activated sludge
		October 16rd	November 20th
Number of control microcosms per sampling time	Sludge + synthetic wastewater	6 microcosms (sizes)	1 microcosm
	E. coli + synthetic wastewater	2 microcosms (duplicate)	
Total number	Experimental microcosms: 42 (16 × three times sampling)<br>Control microcosms: 9 (9 × once sampling)<br>Total microcosms: 51		

**Uptake experiments in 24-well plates**			
Sampling time for both experimental and control microcosms	Three times for triplicate	Different AGS size fractions	Activated sludge
		November 23th<br>December 11th<br>January 29th	December 11th<br>January 20th<br>February 3rd
Number of experimental slides per sampling time	Sludge with spiked *E. coli*	18 slides (6 sizes × 3 slides)	3 slides
Number of control slides per sampling time	Sludge only	6 slides (6 sizes × 1 slide)	1 slide
	E. coli only	1 slide	
Total number	Experimental slides: 63 (21 × three times sampling)<br>Control slides: 24 (8 × three times sampling)<br>Total slides: 87		

Table S6. *Observed protozoan counts on microscope slides and normalized counts based on supernatant volume across six AGS size fractions and activated sludge. Data represent the mean ± standard deviation from triplicate microscopic observations.*

	**Observed counts on the slides (ind)**			**Supernatant-normalized counts (ind mL^{-1})**
**Sludge fraction**	**Sessile ciliate**	**Crawling ciliate**	**Free-swimming ciliate**	**Free-swimming ciliate**
>4 mm	1426 ± 213	2 ± 2	71 ± 6	295 ± 5
2-4 mm	2802 ± 281	1 ± 3	79 ± 14	263 ± 10
1-2 mm	2237 ± 148	1 ± 4	59 ± 2	295 ± 6
0.6-1 mm	649 ± 55	6 ± 6	123 ± 30	341 ± 26
0.2-0.6 mm	352 ± 44	12 ± 11	130 ± 25	361 ± 11
<0.2 mm	362 ± 27	20 ± 19	142 ± 23	394 ± 10
Activated sludge	532 ± 59	139 ± 47	129 ± 22	339 ± 13

Table S7. *Average distribution of six AGS size fractions within AGS reactors in a full-scale AGS plant during one-year sampling periods (May 2023 to April 2024).*

Size range (mm)	Concentration (g SS L^{-1})	Distribution[a] (%)
<0.2	0.89 ± 0.32	10.9 ± 3.4
0.2-0.6	1.65 ± 0.54	20.3 ± 3.1
0.6-1	0.85 ± 0.25	6.9 ± 2.2
1-2	1.31 ± 0.45	17.3 ± 3.2
2-4	2.46 ± 0.75	31.8 ± 3.6
>4	1.56 ± 0.72	12.8 ± 2.4
Total	8.72 ± 0.98	100

[a] The AGS size distribution was calculated as the concentration of a given size fraction divided by the total sludge concentration within a reactor. The average values and standard deviations were determined based on the AGS size distributions from three randomly selected reactors each month over 12 consecutive months.

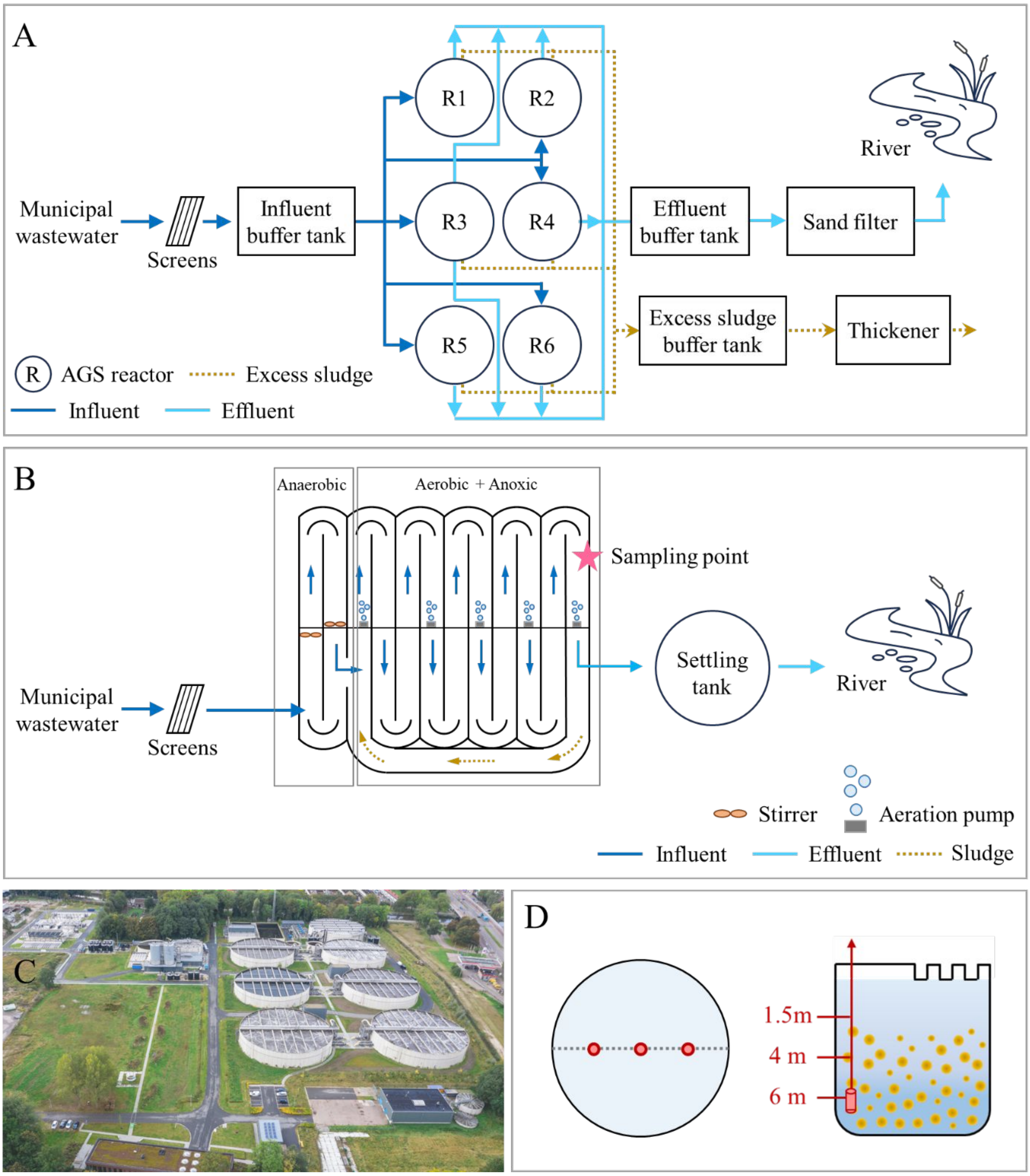

Figure S1. *Schematic diagram showing the treatment process in the full-scale AGS plant in Utrecht (A) and the full-scale activated sludge plant in Bennekom (B); Top view of the Utrecht AGS plant (C); Three sampling points at three varying depths in each AGS reactor (D).*

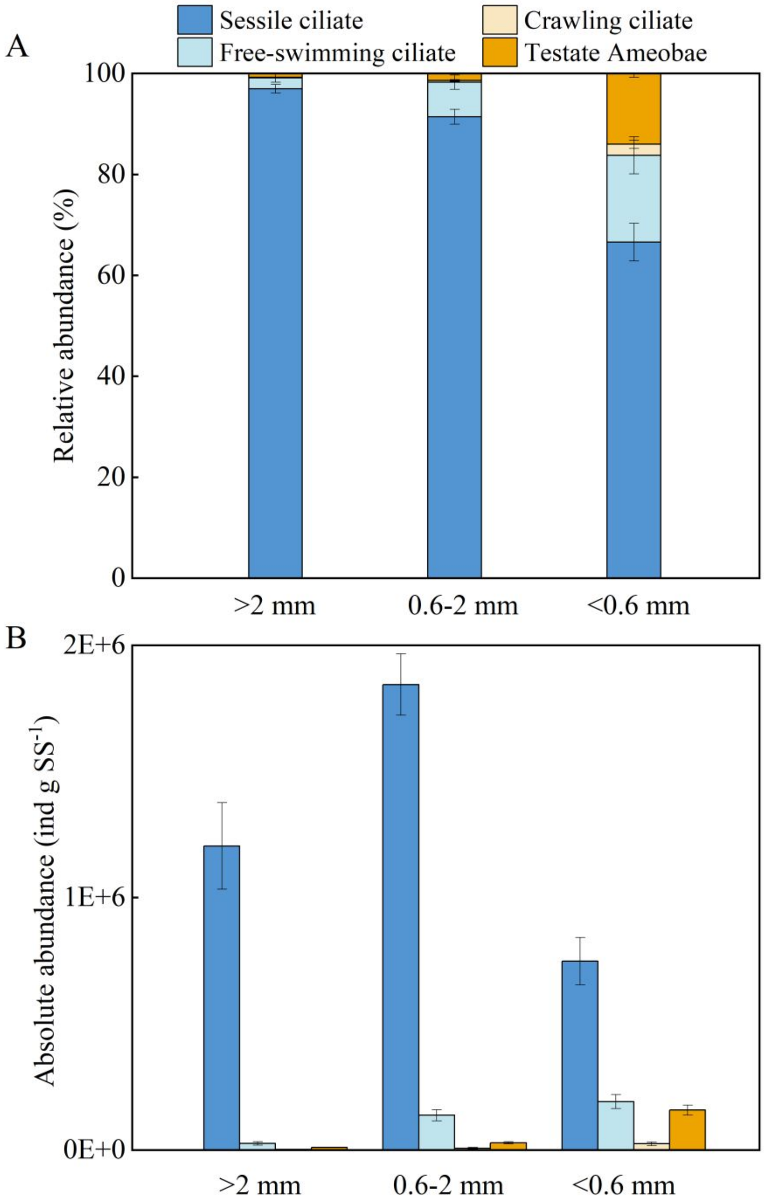

Figure S2. *Relative (A) and absolute abundance (B) of sessile ciliates, free-swimming ciliates, crawling ciliates, and testate amoebae across three AGS size fractions in pre-experiment without wet sieving pretreatment. Absolute abundance refers to the number of individuals counted in sludge samples via microscopy, while relative abundance represents the proportion of each protozoan group relative to the total protozoan count within each size fraction.*

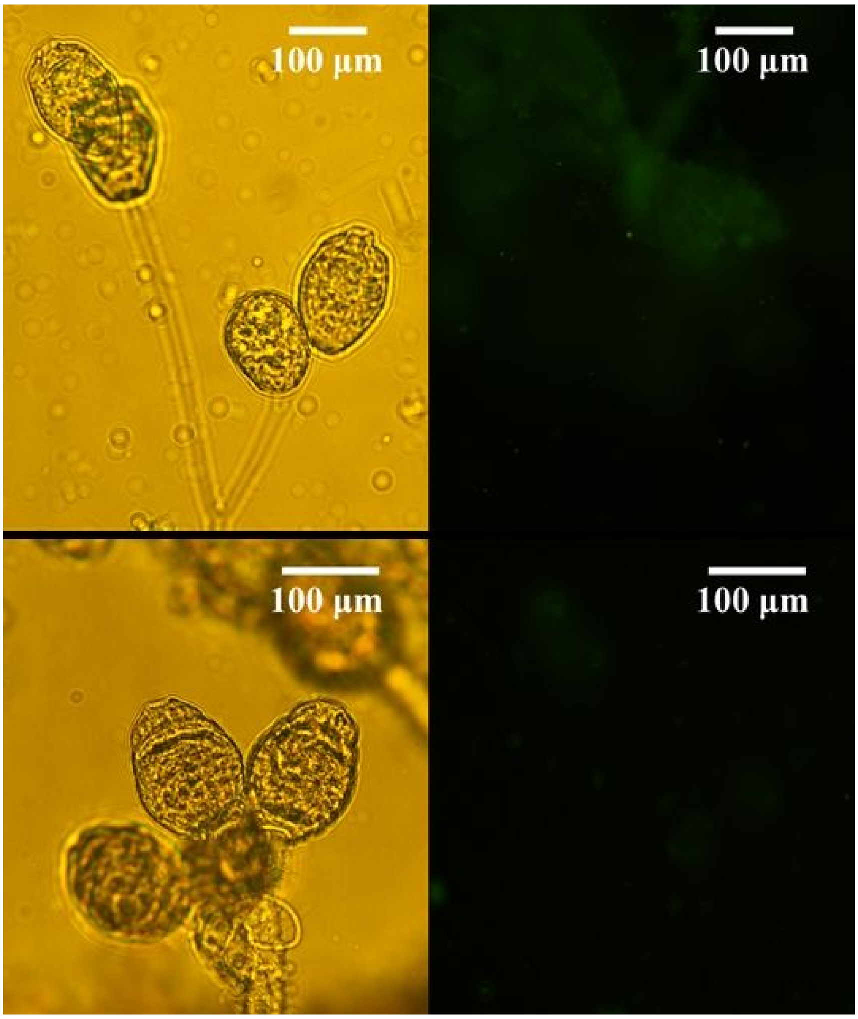

Figure S3. *Immobilization and inactivation of protozoa by 1.6% of paraformaldehyde (PFA). Microscopic images showing protozoa in overlapped fluorescent and bright fields (Left) and the fluorescent field only (Right).*

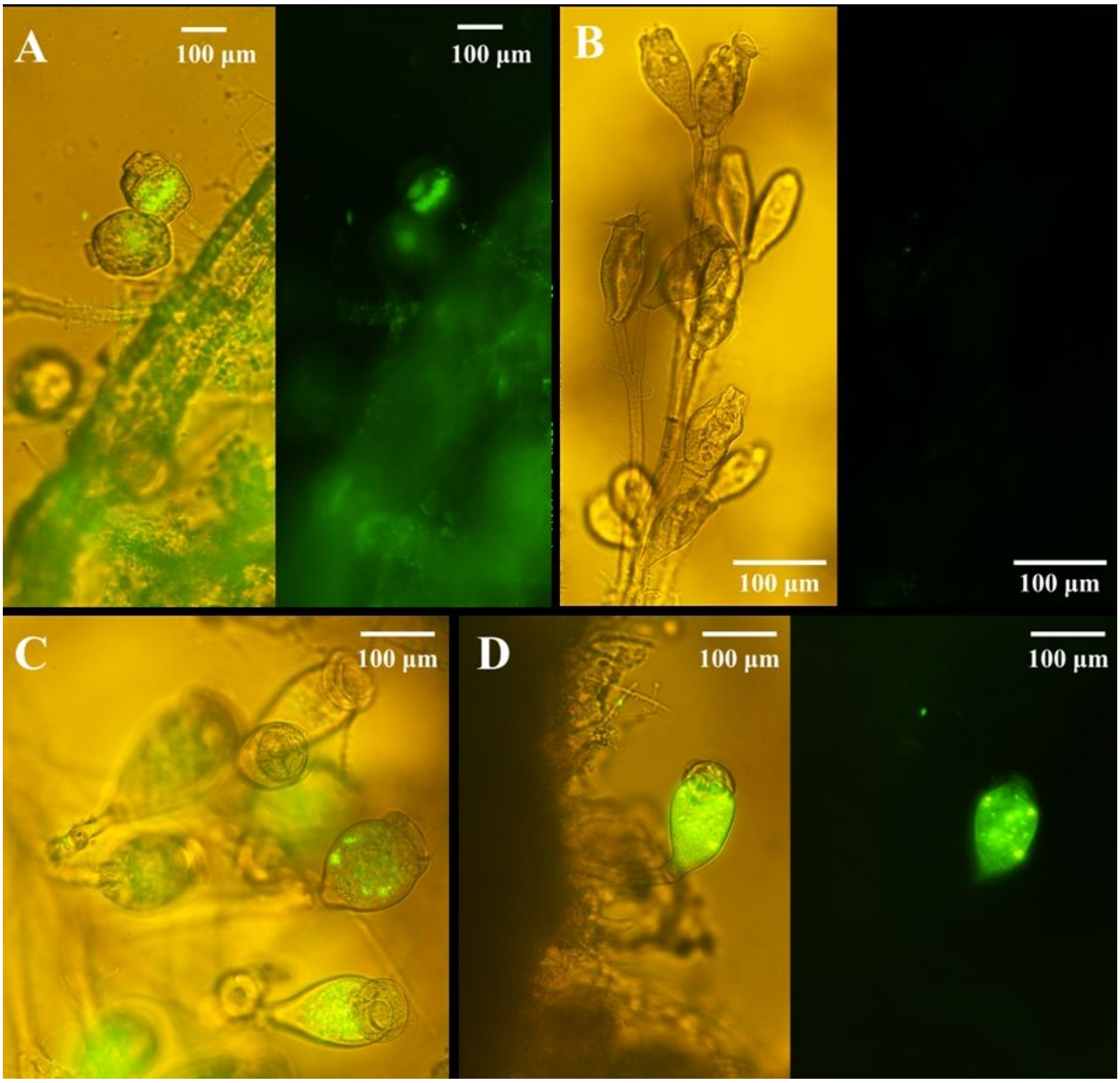

Figure S4. *Uptake behaviour of fluorescent E. coli by protozoa under two inhibitory conditions: Alkaline conditions (A) and low-temperature conditions after 1 hour (B) and 24 hours (C)(D); The left images in (A), (B), and (D) display overlapping fluorescent and bright fields, while the right images show fluorescent field only.*

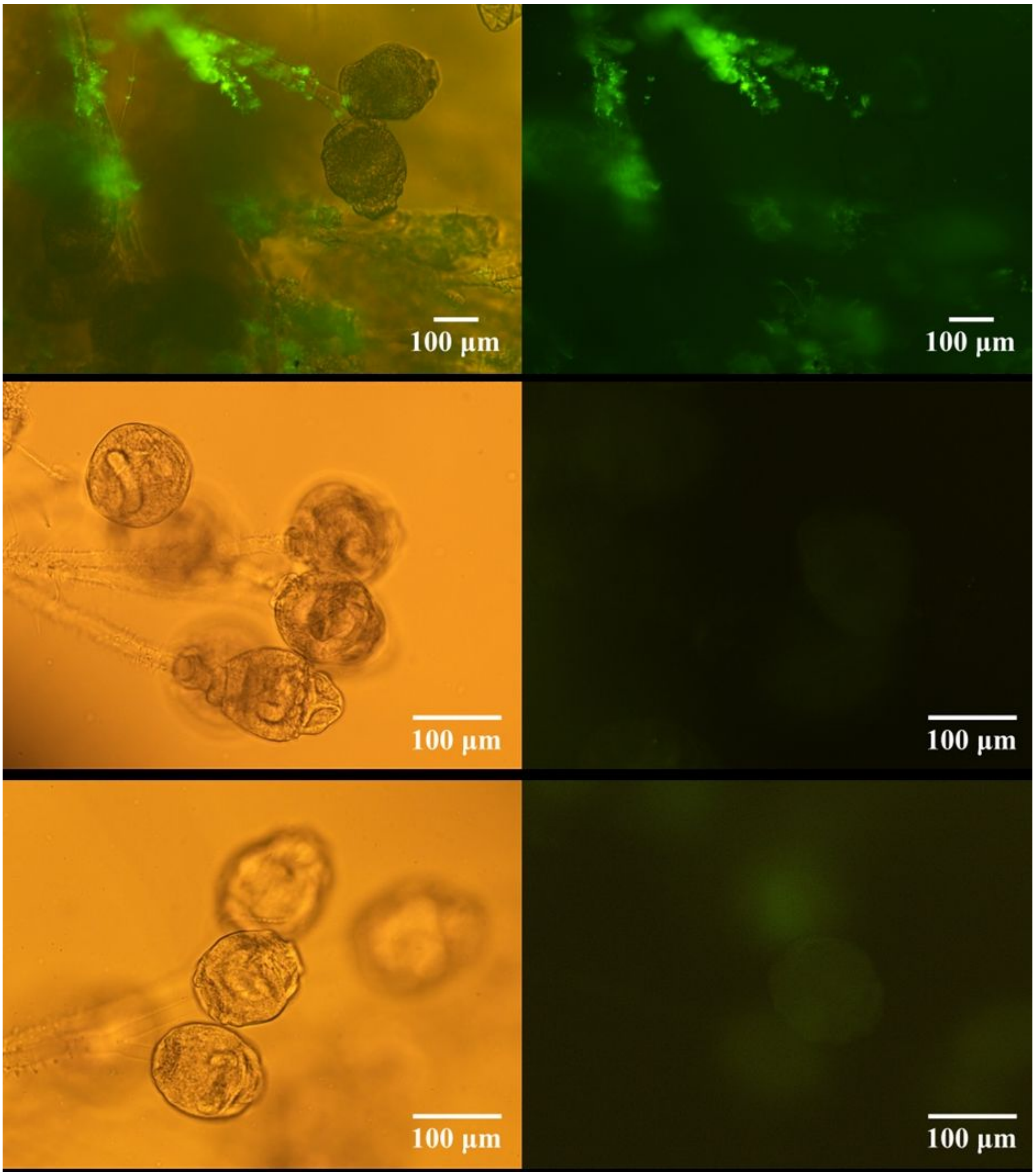

Figure S5. *Uptake behaviour of fluorescent E. coli by protozoa under anoxic conditions after 24 hours; Microscopic images showing protozoa in overlapped fluorescent and bright fields (Left) and the fluorescent field only (Right).*

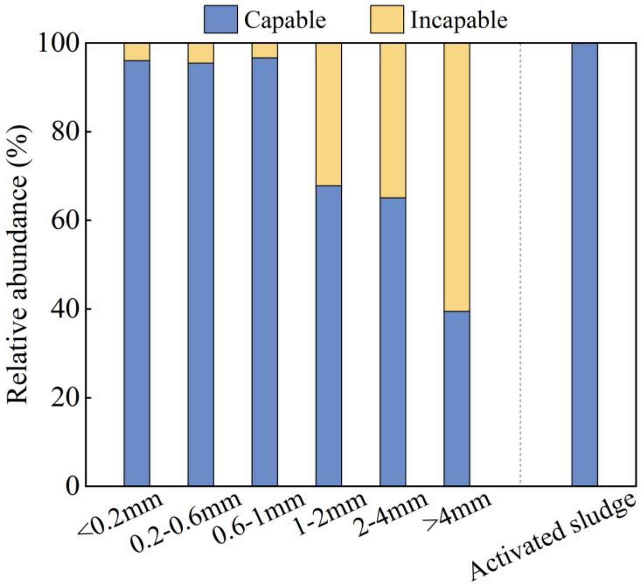

Figure S6. *Relative abundance of Glaucoma capable or incapable of preying on E. coli in six AGS size fractions and activated sludge.*

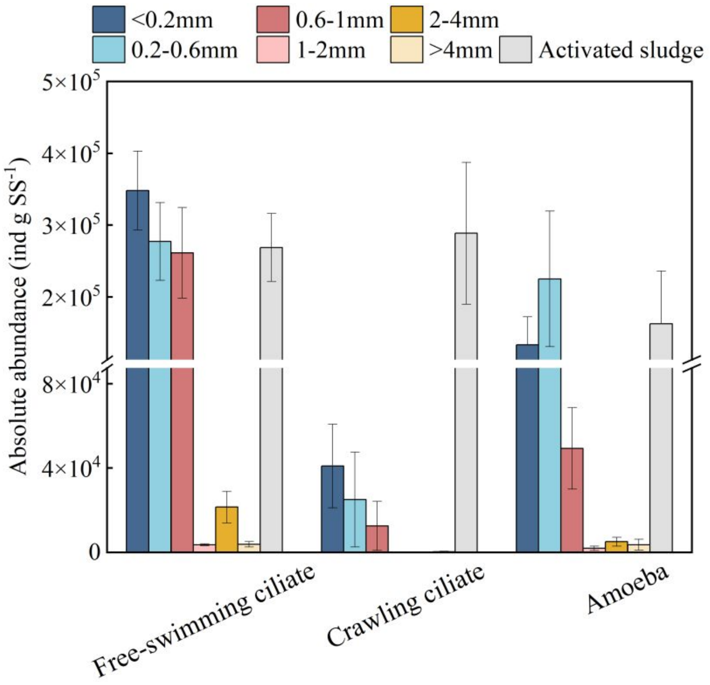

Figure S7. *Absolute abundance of free-swimming ciliates, crawling ciliates, and amoebae in six AGS size fractions and activated sludge.*

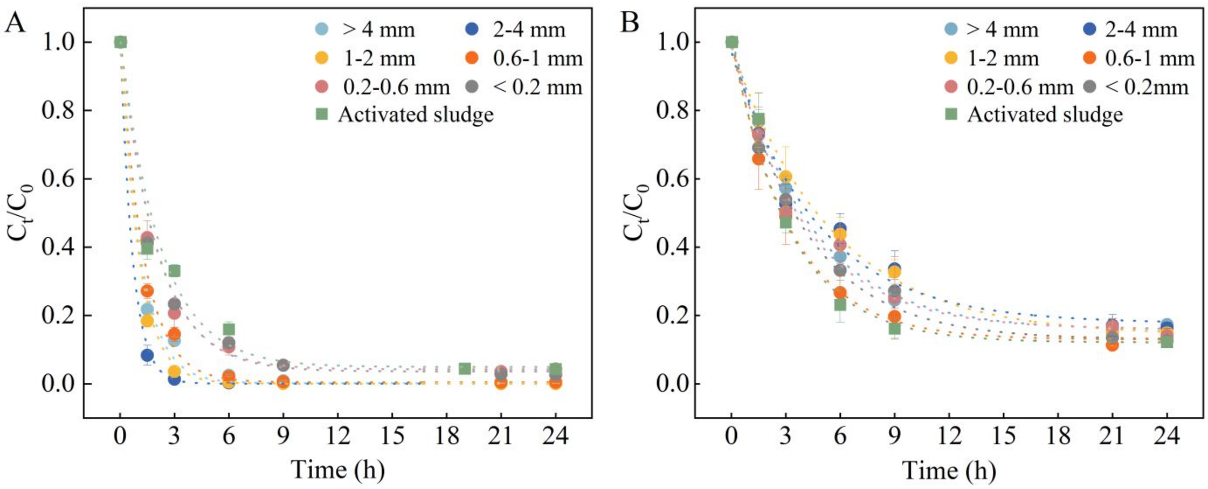

Figure S8. *Residual E. coli in the liquid phase (Ct/C0) over 24 hours in batch bottles with six AGS size fractions and activated sludge under aerobic conditions (combined biological predation, non-predatory biological process, and abiotic process) (A) and anoxic conditions (non-predatory biological and abiotic processes)(B); The value of Ct/C0 was fitted to a first-order bacterial decay model.*

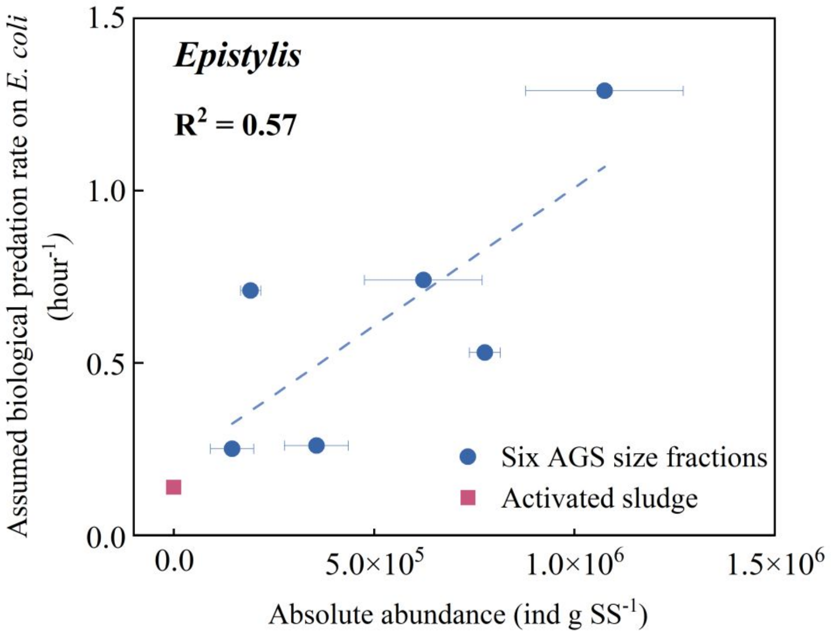

Figure S9. *Linear relationships (blue dotted line) between the absolute abundances of Epistylis and the assumed biological predation rates on E. coli in six AGS size fractions (blue dots) and activated sludge (pink dots). The blue dotted line represents the fit obtained using data from six AGS size fractions, excluding data from activated sludge.*

References

1. Ali, M.; Wang, Z.; Salam, K. W.; Hari, A. R.; Pronk, M.; van Loosdrecht, M. C. M.; Saikaly, P. E., Importance of species sorting and immigration on the bacterial assembly of different-sized aggregates in a full-scale aerobic granular sludge plant. *Environ. Sci. Technol.* **2019**, *53*, 8291-8301. DOI: 10.1021/acs.est.8b07303
2. Toh, S.; Tay, J.; Moy, B.; Ivanov, V.; Tay, S., Size-effect on the physical characteristics of the aerobic granule in a SBR. *App. Microbiol. Biotechnol.* **2003**, *60*, 687-695. DOI: 10.1007/s00253-002-1145-y
3. Alves, O. I. M.; Araújo, J. M.; Silva, P. M. J.; Magnus, B. S.; Gavazza, S.; Florencio, L.; Kato, M. T., Formation and stability of aerobic granular sludge in a sequential batch reactor for the simultaneous removal of organic matter and nutrients from low-strength domestic wastewater. *Sci. Total Environ.* **2022**, *843*, 156988. DOI: 10.1016/j.scitotenv.2022.156988
4. Jang, A.; Yoon, Y. -H.; Kim, I. S.; Kim, K. -S.; Bishop, P. L., Characterization and evaluation of aerobic granules in sequencing batch reactor. *J. Biotechnol.* **2003**, *105*, 71-82. DOI: 10.1016/S0168-1656(03)00142-1
5. Li, Y.; Liu, Y., Diffusion of substrate and oxygen in aerobic granule. *Biochem. Eng. J.* **2005**, 27, 45-52. DOI: 10.1016/j.bej.2005.06.012
6. World Health Organization, WHO integrated global surveillance on ESBL-producing *E. coli* using a "One Health" approach: implementation and opportunities. World Health Organization, Geneva, Switzerland. **2021**.
7. Goulter, R. M.; Gentle, I. R.; Dykes, G. A., Issues in determining factors influencing bacterial attachment: a review using the attachment of Escherichia coli to abiotic surfaces as an example. *Lett. Appl. Microbiol.* **2009**, *49* (1), 1-7. DOI: 10.1111/j.1472-765X.2009.02591.x
8. Nakaya, Y.; Nagahashi, N.; Hirano, R.; Ishizuka, Y.; Satoh, H., Physicochemical parameters affecting the adhesion of ciprofloxacin-resistant Escherichia coli to activated sludge. *Water Sci. Technol.* **2024**, *89* (9), 2457-2467. DOI: 10.2166/wst.2024.134
9. Liu, Y.; Tay, J. -H., The essential role of hydrodynamic shear force in the formation of biofilm and granular sludge. *Water Res.* **2002**, *36*, 1653-1665. DOI: 10.1016/S0043-1354(01)00379-7
10. Berne, C.; Ellison, C. K.; Ducret, A.; Brun, Y. V., Bacterial adhesion at the single-cell level. *Nat. Rev. Microbiol.* **2018**, *16* (10), 616-627. DOI: 10.1038/s41579-018-0057-5
11. Curds, C. R.; Cockburn, A.; Vandyke, J. M., An experimental study of the role of the ciliated protozoa in the activated-sludge process. *Wat. Pollut. Control*, **1968**, *67*, 312-329.
